# tRNA-mediated codon-biased translation in mycobacterial hypoxic persistence

**DOI:** 10.1038/ncomms13302

**Published:** 2016-11-11

**Authors:** Yok Hian Chionh, Megan McBee, I. Ramesh Babu, Fabian Hia, Wenwei Lin, Wei Zhao, Jianshu Cao, Agnieszka Dziergowska, Andrzej Malkiewicz, Thomas J. Begley, Sylvie Alonso, Peter C. Dedon

**Affiliations:** 1 Infectious Disease Interdisciplinary Research Group, Singapore-MIT Alliance for Research and Technology, 1 CREATE Way, #03-14 Enterprise Wing, Singapore 138602, Singapore; 2 Department of Microbiology and Immunology Programme, Yong Loo Lin School of Medicine, 28 Medical Drive, Centre for Life Sciences, Level 3, National University of Singapore, Singapore 117456, Singapore; 3 Department of Biological Engineering, Massachusetts Institute of Technology, 77 Massachusetts Avenue, Cambridge, Massachusetts 02139, USA; 4 Department of Chemistry, Massachusetts Institute of Technology, 77 Massachusetts Avenue, Cambridge, Massachusetts 02139, USA; 5 Institute of Organic Chemistry, Lodz University of Technology, Zeromskiego street 116, 90-924 Lodz, Poland; 6 College of Nanoscale Science and Engineering, State University of New York, 257 Fuller Road, Albany, New York 12203, USA; 7 Center for Environmental Health Sciences, Massachusetts Institute of Technology, 77 Massachusetts Avenue, Cambridge, Massachusetts 02139, USA

## Abstract

Microbial pathogens adapt to the stress of infection by regulating transcription, translation and protein modification. We report that changes in gene expression in hypoxia-induced non-replicating persistence in mycobacteria—which models tuberculous granulomas—are partly determined by a mechanism of tRNA reprogramming and codon-biased translation. *Mycobacterium bovis* BCG responded to each stage of hypoxia and aerobic resuscitation by uniquely reprogramming 40 modified ribonucleosides in tRNA, which correlate with selective translation of mRNAs from families of codon-biased persistence genes. For example, early hypoxia increases wobble cmo^5^U in tRNA^Thr(UGU)^, which parallels translation of transcripts enriched in its cognate codon, ACG, including the DosR master regulator of hypoxic bacteriostasis. Codon re-engineering of *dosR* exaggerates hypoxia-induced changes in codon-biased DosR translation, with altered *dosR* expression revealing unanticipated effects on bacterial survival during hypoxia. These results reveal a coordinated system of tRNA modifications and translation of codon-biased transcripts that enhance expression of stress response proteins in mycobacteria.

## Abstract (web summary)

Mycobacteria can adapt to the stress of human infection by entering a dormant state. Here the authors show that hypoxia-induced dormancy in *M. bovis* BCG involves the reprogramming of tRNA wobble modifications and copy numbers, coupled with biased use of synonymous codons in survival genes.

## Graphical abstract

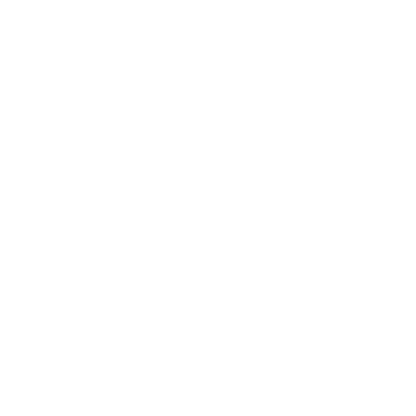

All cells respond to environmental changes by regulating gene expression at multiple levels. Among human pathogens, mycobacteria have evolved a genetically programmed mechanism of adapting to the stress of human infection by entering a quiescent state in which cell replication is halted or slowed in response to nutrient deprivation, hypoxia and other stresses encountered in the granulomas that characterize tuberculosis^1^,^2^,^3^. The mechanisms regulating the hypoxic response in mycobacteria have been extensively studied in members of the *Mycobacterium tuberculosis* (Mtb) complex, including the Mtb surrogate, *M. bovis* BCG. For example, hypoxia has been shown to modulate a transcriptional regulatory network that is predictive of changes in lipid metabolism caused by hypoxia^4^. The modest predictive power of transcriptionally based models is likely due to the well-established poor correlation between levels of transcripts and proteins^5^ and points to the potential for translational regulatory mechanisms contributing to cell phenotype.

Here we show how translational mechanisms play an important role in the mycobacterial response to hypoxia. The basis for these studies lies in our observations in budding yeast, in which stress-specific alterations in dozens of modified ribonucleosides in transfer RNA (tRNA) coordinately regulate selective translation of codon-biased messenger RNAs (mRNAs) from families of stress response genes^6^,^7^,^8^,^9^,^10^. There is emerging speculation for the existence of a ‘code of codons' based on gene-specific codon usage patterns^6^,^11^,^12^ that can regulate translation^10^,^13^,^14^,^15^. Among possible mechanisms linking environmental changes to codon-biased translation, recent studies have shown that the dozens of modified ribonucleosides in tRNA form a dynamic system that responds to cellular stress^6^,^7^,^8^,^16^. We have shown that stress-specific alterations in tRNA wobble modifications, which can expand or limit tRNA decoding capabilities^17^,^18^,^19^, facilitate decoding of cognate codons that are over- or under-used in mRNAs, which enhances translational elongation and leads to the selective up- and downregulation of the codon-biased genes^10^,^20^. Given that these mechanisms have yet to be established in prokaryotes, much less shown to play any role in microbial pathogenesis, we identified a role of tRNA reprogramming and selective translation of codon-biased survival proteins in the hypoxia-induced non-replicative state of mycobacteria.

## Results

### Hypoxia reprogrammes tRNA modifications in mycobacteria

**Figure 1:**
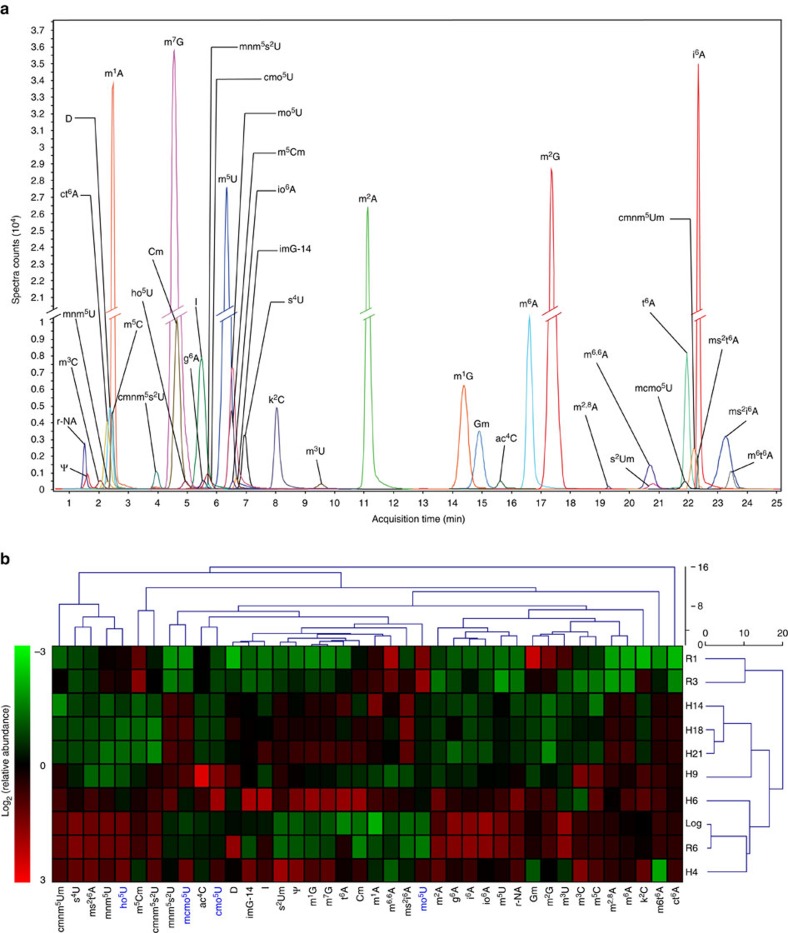
Dynamics of tRNA modifications as BCG enter and exit hypoxic non-replicating persistence. (**a**) Composite extracted ion chromatogram of 40 modified ribonucleosides in BCG tRNA. Full names, structures and LC-MS/MS parameters can be found in Supplementary Data 1. (**b**) Hierarchical clustering analysis of changes in the relative levels of BCG tRNA modification induced by hypoxia (H) on day 0 (Log), 4, 6, 9, 14 and 18, and re-aeration (R) on day 19 (R1), 21 (R3) and 24 (R6) (time course in Supplementary Fig. 1). Hierarchical clustering was performed on mean-centred data (*n*=6) and visualized as a heat map of log_2_ fold-changes relative to Log cultures with colour intensities subjected to standardization by RNA modification: 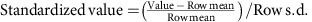 Relative quantification of tRNA modifications can be found in Supplementary Data 2. Modified ribonucleosides ho^5^U, mo5U, cmo^5^U and mcmo^5^U relevant to the discussion in the text are highlighted in blue.

We began this mechanistic analysis by characterizing the full repertoire of tRNA modifications in *M. bovis* BCG and their behaviour under hypoxic stress. Using chromatography-coupled mass spectrometry (LC-MS)^21^,^22^,^23^, we identified 40 distinct ribonucleoside modifications in purified tRNA (Fig. 1a, parameters in Supplementary Data 1). Next, we used dynamic multiple reaction monitoring (MRM; Supplementary Data 1)^22^ to quantify changes in the levels of these modifications as BCG entered and exited a non-replicating, persistent state in a Wayne-like gradual hypoxia model of non-replicating persistence (Supplementary Fig. 1a)^24^. The measurements of tRNA modifications proved to be very precise, with variance among biological replicates differing by an average coefficient of variation of 17±3.8% while differences in modification levels between time points varied with a coefficient of variation of 74±40% (Supplementary Data 2). This validates the rigour of the method for quantifying significant hypoxia-induced changes in tRNA modification levels. Hierarchical clustering analysis of fold-change values for each modification (Fig. 1b, Supplementary Data 2) distinguished the three classical phases of hypoxia-induced persistence: aerated growth and non-replicating persistence stages 1 and 2 (Supplementary Fig. 1a). However, this analysis of fold-change values on days 0–21 of hypoxia (Log, H4–21) and days 1–6 of re-aeration (R1–6) also distinguished two transitional phases at the entry (H4) and exit (R1–3) of hypoxic bacteriostasis (Fig. 1a, Supplementary Fig. 1a). We termed these phases hypoxic transition (HT) and early resuscitation (ER), respectively (Supplementary Fig. 1a). Hypoxia thus induced distinct patterns of change in the 40 tRNA modifications in BCG, patterns that predicted the stages of hypoxic persistence and resuscitation.

### Hypoxia regulates tRNA modifications and tRNA isoacceptors

**Figure 2:**
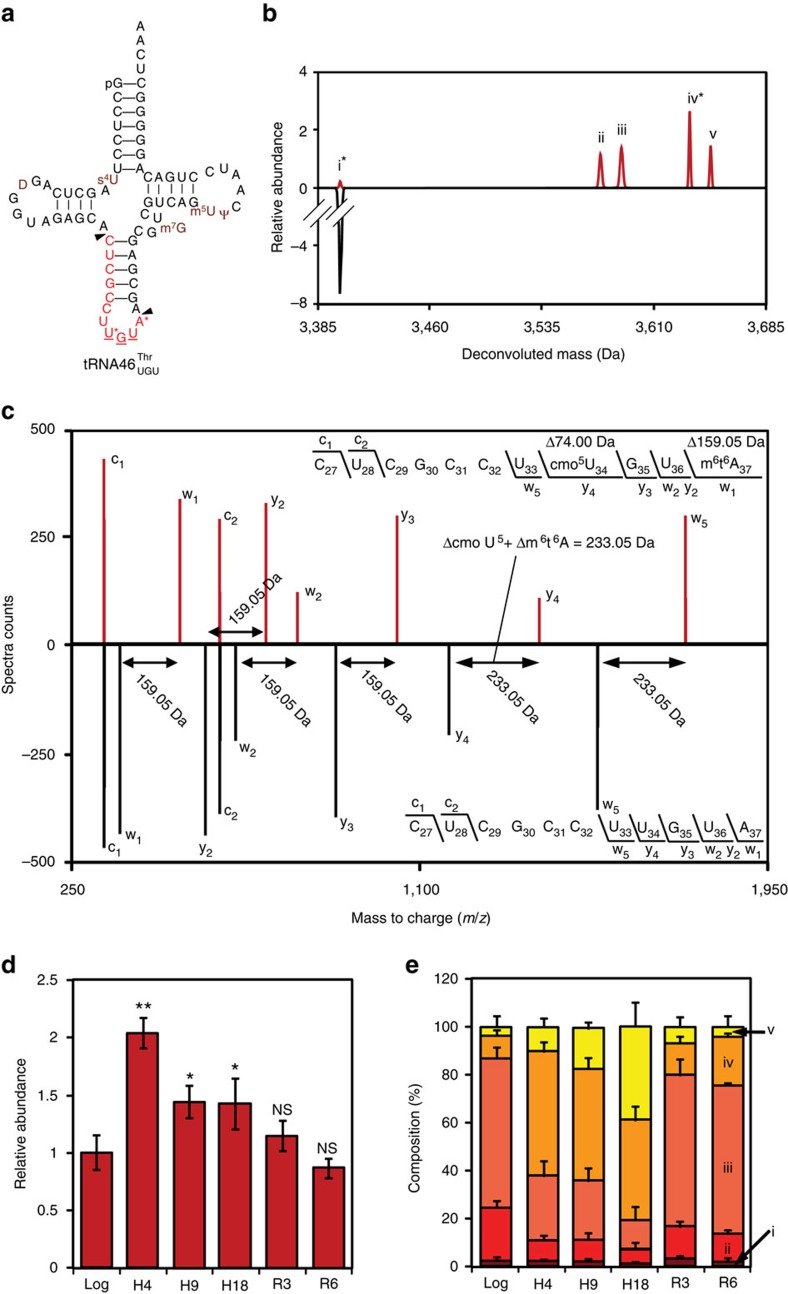
Hypoxia induces tRNA^Thr(UGU)^remodelling. Total tRNA was digested with RNase U2 to generate oligoribonucleotides containing unique fragments from (**a**) tRNA46^Thr(UGU)^ (indicated by arrows) for LC-MS modification mapping and copy number quantification. Positions of conserved 4-thiouridine (s^4^U), dihydrouridine (D), pseudouridine (Ψ), 7-methylguanosine (m^7^G), 5-methyluridine (m^5^U), wobble uridine (U* in **a**) and adenosine37 (A*) are shown in brown. (**b**) Identification of wobble occupancy variants. Maximum entropy deconvolution of MS spectra for H9 tRNA hydrolysate (top—red trace) and a synthetic standard of UCGCCUUGUA (bottom—black trace). The peaks were identified as follows: i—CUCGCCUUGUA, ii—CUCGCCUho^5^UGUm^6^t^6^A, iii—CUCGCCUmo^5^UGUm^6^t^6^A, iv—CUCGCCUcmo^5^UGUm^6^t^6^A and v—CUCGCCUmcmo^5^UGUm^6^t^6^A by *de novo* sequencing. (**c**) Representative targeted fragmentation of peak iv for *de novo* sequencing. Mirror plot shows resolved isotope deconvoluted MS/MS spectra of the oligonucleotide CUCGCCUcmo^5^UGUm^6^t^6^A (top—red trace) and synthetic standard UCGCCUUGUA (bottom—black trace). The 159.05 Da mass shifts in w_1_, w_2_, y_2_ and y_3_ ions are consistent with m^6^t^6^A_37_. The 233.05 Da mass shifts in y_4_ and w_5_ ions are consistent with the sum of m^6^t^6^A_37_ (Δ159.05 Da) and cmo^5^U_34_ (Δ74.0 Da) modifications. The oligonucleotide sequence is denoted in standard ion fragmentation nomenclature on the top right. Deconvoluted masses for peaks i–v and validated fragment ions are available in Supplementary Data 3. (**d**) Fold-changes in tRNA^Thr(UGU)^ copy numbers at H4, H9, H18, R3 and R6 against Log. tRNA7^Thr(UGU)^ was present at 943 (±217) copies per CFU under Log conditions as determined by selected reaction monitoring (Supplementary Fig. 2). Data represent mean±s.e.m.; *n*=4. Statistical analysis by one-way analysis of variance (ANOVA) with Dunnett's test versus Log: NS, not significant; *P*<0.05 and *P*<0.01 are denoted as * and ** respectively. (**e**) Composition of the tRNA^Thr(UGU)^ pool in terms of its wobble occupancy variants (identified in peaks i–v), at indicated time points expressed as percentages of their sum total.

We next explored the link between hypoxia-altered modification patterns in the tRNA population and translation of codon-biased genes by defining hypoxia effects on wobble modifications in individual tRNA species. For example, levels of 5-oxyacetyl-uridine (cmo^5^U), noted for its ability to decode G-ending codons^25^,^26^, were found to increase by >350% in early hypoxia in total tRNA (Fig. 1b; Supplementary Data 2). In *Escherichia coli*, CmoB converts 5-hydroxyuridine (ho^5^U) to either 5-methoxyuridine (mo^5^U) or cmo^5^U depending upon the availability of S-carboxymethyl-S-adenosylmethionine (carboxy-SAM) produced from SAM by CmoA^27^. CmoM then methylates cmo^5^U to form mcmo^5^U (ref. 28). While this biosynthetic pathway has not been annotated in BCG, we found homologues of *cmoA* and *cmoB* in BCG: BCG_0612 (55% coverage of CmoA, *E* value=4e^−4^ by blastp) and BCG_2975c (51% coverage of CmoB, *E* value=2e^−5^ by blastp). In addition to finding ho^5^U, mo^5^U, cmo^5^U and mcmo^5^U in BCG tRNA (Fig. 1b)^8^, mo^5^U has been mapped to the wobble position of tRNA^Thr(UGU)^ in *Bacillus subtilis*^29^ while mcmo^5^U and cmo^5^U have been mapped to the same position in *E. coli* tRNA^Thr(UGU)28^. Building on these observations, we defined the wobble occupancy of tRNA^Thr(UGU)^ in BCG and measured hypoxia-induced changes in the levels of 3 tRNA^Thr^ isoacceptors in BCG by mass spectrometric sequencing and quantification of RNase U2-generated oligonucleotides (Fig. 2, Supplementary Fig. 2, Supplementary Data 3)^30^. There was a significant decrease in wobble ho^5^U in tRNA^Thr(UGU)^ at H4 accompanied by a nearly complete shift from mo^5^U under aerobic conditions to cmo^5^U and mcmo^5^U at H4 and H9 (Fig. 2e). This could reflect a hypoxia-induced increase in CmoA-catalysed formation of carboxy-SAM to shift CmoB activity from mo^5^U to cmo^5^U; proteomic analyses discussed shortly support this model. This hypoxic increase in wobble cmo^5^U in tRNA^Thr(UGU)^ was accompanied by a significant increase in number of copies of this tRNA (Fig. 2d) and decreases in the levels of the other two Thr tRNA isoacceptors (Supplementary Fig. 2). All the changes reverted to Log levels during aerobic resuscitation (Fig. 2d,e; Supplementary Fig. 2). These results are consistent with coordinated up- and downregulation of specific tRNA wobble modifications and tRNA isoacceptor levels in response to hypoxia, which raises the question of a link to hypoxia-induced translation of mRNAs with biased use of the cognate codons for these tRNAs.

### Biased codon usage across the BCG genome

We next tested the hypothesis that the BCG genome was organized with biased use of synonymous codons, with codon biases linking persistence-related genes to hypoxia-reprogrammed tRNAs. Using a gene-specific codon counting algorithm^31^, we analysed codon usage patterns in the 3,951 protein-coding genes in BCG, with the heat map shown in Supplementary Fig. 3 revealing two groups of genes with significantly biased use of synonymous codons. In the 91 genes in Group 1, 21 members of the 48-gene Dos regulon, which controls the early hypoxic response^4^,^32^, were significantly biased with C- and G-ending codons, including higher than average ACG usage and lower than genome average ACC usage. Of these, nine *dosR* regulon genes show significant ACG/ACC biases after correction for gene length and global codon bias by z-transformation: BCG_0113, BCG_0114, BCG_0614, BCG_0615 (*nrdZ*), BCG_1772, BCG_1773c, BCG_2024c (*fdxA*), BCG_2013, and BCG_3156c (*dosR*). Considering that there are 3,952 genes in the BCG genome, the probability of picking the same nine genes with this exact feature from the two data sets by random chance is 7.5 × 10^−8^. As illustrated in Supplementary Fig. 3b, the gene for *dosR*, the transcription factor regulating activity of the Dos regulon, was found to be highly significantly enriched in Thr^ACG^ codons and impoverished in Thr^ACC^ codons.

These codon biases, along with the fact that G-ending codons in 4-fold degenerate codon boxes (Leu, Val, Ser, Pro, Thr, Ala) are read by modified wobble uridines (Supplementary Fig. 4), become important in light of the observation that hypoxia leads to the upregulation of wobble cmo^5^U in tRNA^Thr(UGU)^. Indeed, cmo^5^U is noted for its ability to decode G-ending codons^25^,^26^. Similarly, the putative CmoA homologue, BCG_0612, is heavily biased in ACA usage (*Z*=2.75, *P*<0.01)—a rare codon decoded solely by tRNA^Thr(UGU)^ and its modified derivatives (Supplementary Fig. 4) and preferably read during the hypoxic transition (Fig. 2). Thus, the BCG genome contains sets of genes with biased use of codons that are read by hypoxia-reprogrammed tRNAs, which raises the question of selective translation of proteins from these genes during hypoxia.

### tRNA modifications are linked to global protein expression

**Figure 3:**
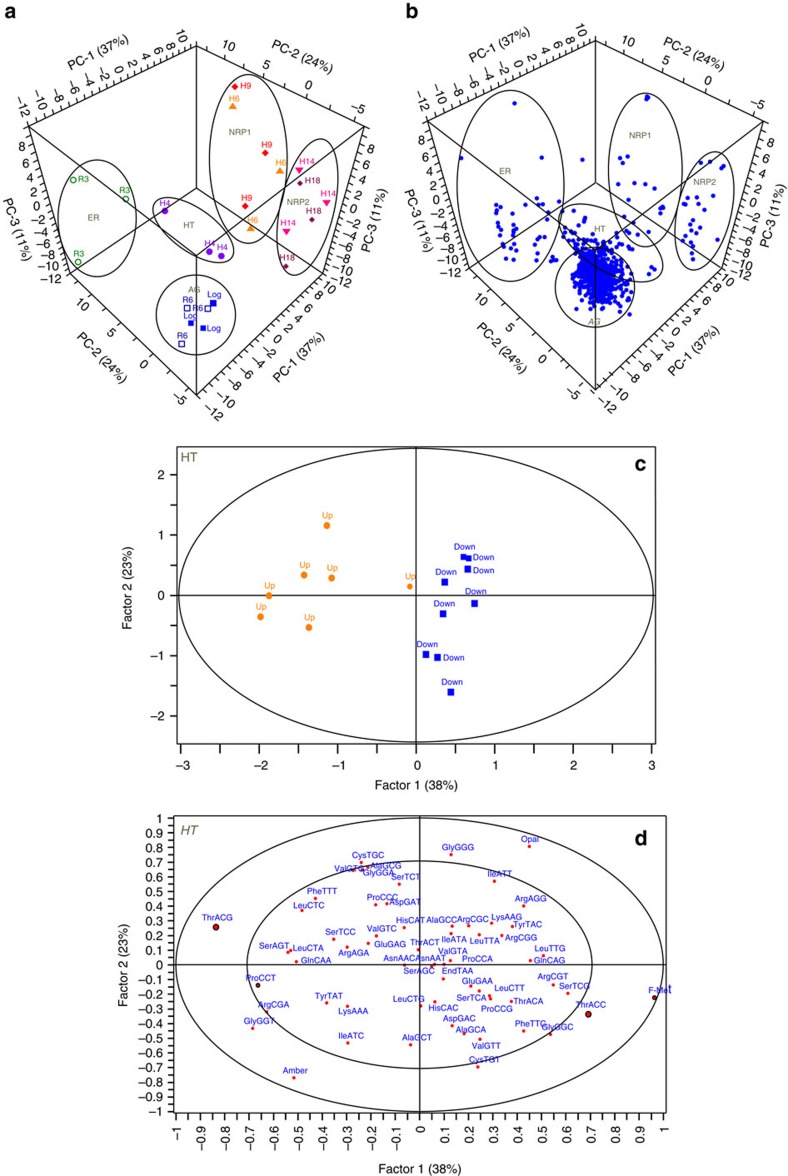
The choice between codons Thr^ACG^ and Thr^ACC^ influences protein up- or downregulation in the BCG response to hypoxia. (**a**,**b**) Principal component analysis was performed with fold-change data for the 965 most quantifiable proteins (loadings, **b**) across the hypoxia time course (scores, **a**). Clustering of sample eigenvectors (Log■, H4·, H6▲, H9⧫, H14▼, H18*, R3○ and R6□) in the scores plot (**a**) and protein variables (Blue filled circle) in the loadings plot (**b**) is highlighted using data eclipses, with the clusters reflecting both the growth phenotypes (acronyms defined in Supplementary Fig. 1a) and the tRNA modification clustering in the heat map in Fig. 1b. Seventy-two per cent of observed variance can be explained by three principal components (*n*=3, PC-1: 37%, PC-2: 24%, PC-3: 11%). (**c**,**d**) Partial least squares regression analysis of significantly up- (Orange filled square) or down- (Blue filled circle) regulated proteins and their codon usages (Red filled circle) at H4 visualized by scores (**c**), and X,Y correlation loadings representing codon usage (X) against extent of protein up- or downregulation (Y) (**d**). The proteins were selected blindly for PLS analysis based on statistical significance (*P*<0.05) and >2 fold-change (*n*=3; unpaired, two-tailed *t*-test; Supplementary Fig. 6a). Ellipse in **c** represents the Hoteling T^2^ limit at *P* value of 0.05 (F-test), while the ellipses in **d** indicate the explained variance. Outer and inner ellipses indicate 100% and 50% explained variance, respectively. Codons contributing significantly to the regression (cross validation, by Marten's uncertainty test) are circled in black. 61% of observed variance can be explained by two latent factors (*n*=3, Factor-1: 38%, Factor-2: 23%). AG, aerated growth; ER, early resuscitation; HT, hypoxic transition; NRP, non-replicating persistence (stages 1 and 2); PLS, partial least square.

To test the hypothesis that the changes in the hypoxia-remodelled tRNA^Thr^ pool correlate with translation of codon-biased mRNAs to affect expression of Dos regulon and other proteins, we performed iTRAQ proteomic analyses to compare changes in protein levels across the hypoxic time course at Log, H4, H6, H9, H14, H18, R3 and R6. By cross-validating protein identifications between SpectrumMill and X!tandem, we were able to match 122,546 spectra to 36,751 unique peptides that mapped onto 2,455 proteins (62% proteome coverage at 4.9% peptide FDR; Supplementary Fig. 5a,b). Of these, 965 proteins could be consistently quantified by two or more peptides in all samples across the time course (*P*<0.01; protein FDR<0.01%; no missing values) (Supplementary Data 4), with the proteins distributed across all major gene ontology categories (Supplementary Fig. 5d). A scores plot from a principal component analysis (Fig. 3a) revealed clustering of time points from the hypoxic time course, with the clustering precisely recapitulating the five stages of hypoxia-induced persistence and resuscitation defined earlier by the tRNA modifications (Fig. 1b) and hypoxic growth curve (Supplementary Fig. 1a). This clustering behaviour was driven by groups of highly co-varying proteins that defined each time point and stage, as shown in the loadings plot of Fig. 3b.

A mechanistic link between tRNA modification changes and changes in protein expression appears to arise in another striking feature that emerged from a partial least squares regression analysis of the most significantly up- or downregulated proteins (>2-fold change, *P*<0.05; Supplementary Fig. 6; Supplementary Data 4) and the codon usage patterns in their genes: pairs of synonymous codons were significantly over- or under-represented in the highly up- and downregulated proteins across the hypoxia time course. For example, after 4 days of hypoxia (H4), the genes for upregulated proteins showed a highly significant preference for ACG over ACC and those for downregulated proteins reversed this usage (0.83<*R*^2^<0.91 for Factor-1, Fig. 3c,d). This behaviour was recapitulated at other hypoxia time points, with codon choices between the synonymous pairs Asn^AAT^/Asn^AAC^, Asp^GAT^/Asp^GAC^, Cys^TGT^/Cys^TGC^, His^CAT^/His^CAC^, Lys^AAA^/Lys^AAG^ and Tyr^TAT^/Tyr^TAC^ distinguishing highly up- or downregulated proteins (Supplementary Fig. 7). The link to tRNA modification changes is illustrated at H4 by the highly correlated increase in wobble cmo^5^U in the tRNA^Thr(UGU)^ that reads ACG, the significant increase in the number of copies of tRNA^Thr(UGU)^ (Fig. 2d), and decreases in the levels of the other two Thr tRNA isoacceptors, including the tRNA^Thr(GGU)^ that reads ACC (Supplementary Fig. 2). These results support a model of stress-induced concerted reprogramming of tRNA modifications and translation of codon-biased mRNAs to affect phenotypic changes.

### The translation model in the context of gene expression

One question that emerges at this point is the extent to which transcriptional pressures and mRNA levels drive codon-biased translation. One could envision an extreme scenario in which hypoxia induces up- and downregulation of only those transcripts that possess the observed stage-specific codon biases in up- and downregulated proteins, with the tRNA reprogramming merely reflecting pressures for translating on ribosomes loaded with these codon-biased transcripts—a purely transcription-driven mechanism. Here we demonstrate that this is not the case in hypoxic BCG based on two different pieces of evidence. First, we note that DosR protein levels significantly diverge from changes in *dosR* transcript levels, with mRNA levels dropping rapidly after H4 and maximal levels of DosR reached at H9 and maintained until re-aeration (post-H18; Supplementary Fig. 5e). In contrast, changes in levels of tRNA^Thr(cmo5UGU)^ closely paralleled DosR expression (Supplementary Fig. 5e). Similarly, protein concentrations of putative CmoA homologue that synthesizes cmo^5^U in BCG increased up to 6.5-fold in early hypoxia (Supplementary Fig. 5e), which is consistent with the shift from wobble mo^5^U to cmo^5^U in tRNA^ThrUGU^ (Fig. 2d).

In a second more generalized analysis, we asked whether codon usage affects protein expression of co-transcribed genes in BCG operons. Since transcription of polycistronic mRNAs from operons in the mycobacterial genome leads to co-transcription of many genes, one would expect to see correlated translational up- and downregulation for all gene partners on an operon and no codon usage differences among translated proteins unless all operonic gene partners shared the same codon usage patterns. To test this idea, we mapped 229 of the 965 proteins detected in our analysis onto 86 operons with polycistronic mRNAs. Next, we compared the protein expression of these genes and their co-transcribed neighbours. In 254 pair-wise comparisons, 73 gene pairs show strong positive correlations (0.4≤*R*^2^≤1) while 45 pairs are anti-correlated (−1≤*R*^2^≤−0.4). Interestingly, on tallying the codon usage differences between correlated and anti-correlated pairs, we note that disproportionate use of Thr^ACG^ in one member of a gene-pair relative to its neighbour is a contributor to anti-correlated protein expression (Supplementary Fig. 8a). Gene pairs with one partner biased in using Thr^ACG^ are 4.6 times more likely (confidence interval: 2.7–8.1, *P*<0.0001) to be anti-correlated in protein expression (Supplementary Fig. 8b). These results highlight the subtle but pervasive influence of synonymous codon usage on gene expression and support the idea of a link between stress-induced tRNA reprogramming and selective translation of codon-biased stress response proteins.

### Codon re-engineering supports codon-biased translation

**Figure 4:**
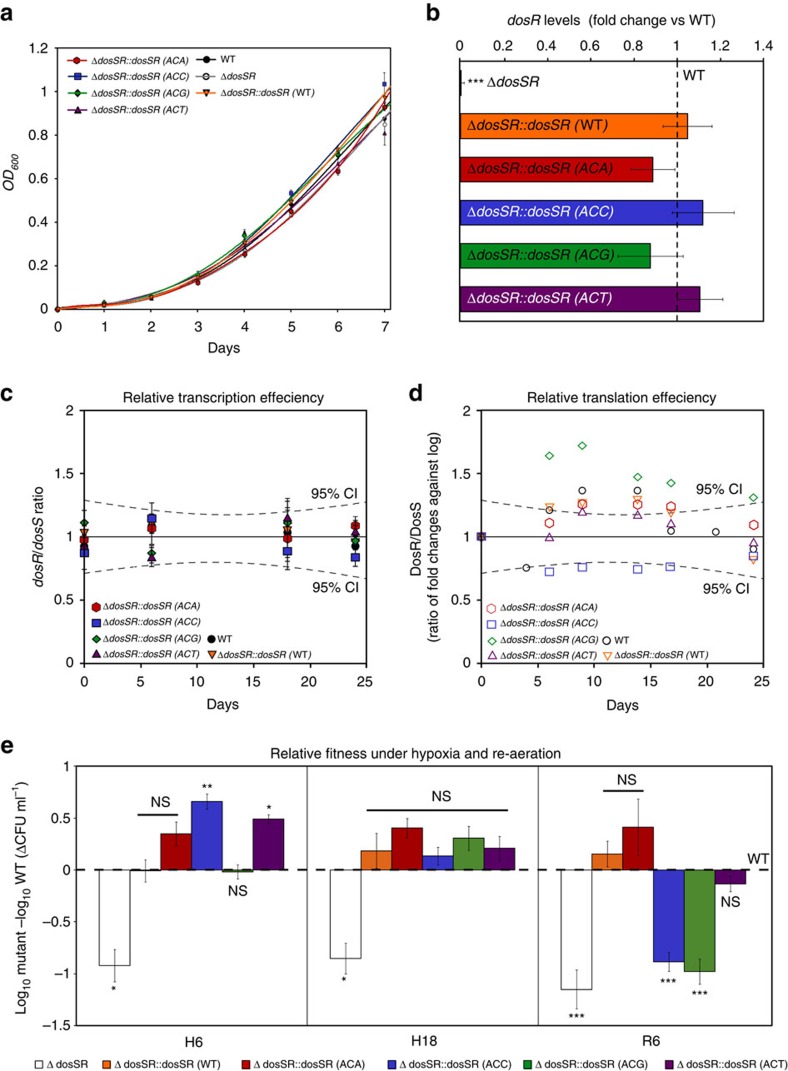
*dosR* mutants re-engineered to use synonymous Thr codons show altered *dosR* and *hspX* expression as well as altered growth phenotypes. Notations used for each strain are explained in the main text, Supplementary Fig. 9a and Supplementary Table 3. (**a**,**b**) Strains had similar growth profiles (**a**), and *dosR* mRNA levels under aerobic conditions (**b**), except for Δ*dosSR*, which served as a negative control (*n*=5; one-way analysis of variance (ANOVA) with Tukey's HSD). The dashed line in **b** shows mean *dosR* expression in wild-type (WT) BCG. (**c**,**d**) Relative efficiencies of *dosR* transcription relative to *dosS* (**c**) and DosR translation relative to DosS (**d**) in Thr codon-engineered mutants. The dashed lines show the 95% confidence boundary for *dosR*/*dosS* ratios being *y*=1 (by analysis of covariance (ANCOVA)); Ratios of fold-changes in DosR to those of DosS at the same time point that go beyond this 95% confidence boundary are diverging significantly from what would be predicted from their mRNA levels. Protein levels of DosR and DosS determined by SRM (Supplementary Fig. 10) tabulated in Supplementary Data 3. (**e**) Differential fitness of *dosR* mutants relative to WT at early hypoxia (H6), late hypoxia (H18) and post recovery (R6). Statistical treatments: *n*≥5; mean±s.e.m.; NS, not significant; significance at *P*<0.05, *P*<0.01 and *P*<0.001 are denoted as *, ** and ***, respectively, as determined by two-way ANOVA with Bonferroni post-tests (versus Log) considering interactions between mutations and hypoxia. CI, confidence interval.

To further ascertain the importance of codon usage patterns in translation efficiency and survival in the BCG hypoxic response, we generated a series of recombinant BCG strains with *dosR* engineered to use only one of four possible Thr codons: ACA, ACC, ACG or ACU (Supplementary Fig. 9a). These are termed *ΔdosSR*::*dosSR(ACA) ΔdosSR*::*dosSR(ACC), ΔdosSR*::*dosSR(ACG)* and *ΔdosSR*::*dosSR(ACT),* respectively; note that *dosS* retains wild-type (WT) codon usage in all constructs. *dosS* and *dosR* belong to the same operon and RNAseq analysis confirmed that they are co-transcribed on the same mRNA (GEO accession study GSE66883). In aerobic exponential phase growth, these synonymous Thr codon mutations were silent in terms of growth rate (Fig. 4a), *dosR* transcription (Fig. 4b), the ratio of transcription of *dosR* to its operon partner, *dosS* (Fig. 4c), and translation (relative to *dosS*) (Fig. 4d). Knocking out both genes (*ΔdosSR*) is deleterious for mycobacteria during hypoxia and upon re-aeration (Fig. 4e, white bar), but their restoration in the *ΔdosSR*::*dosSR(WT)* construct restored fitness (Fig. 4e orange bar; Supplementary Fig. 9b)^33^. However, upon induction of hypoxia, the translational efficiency of DosR in the synonymous Thr codon mutants is maximally enhanced by ACG and maximally reduced by ACC during hypoxia (Fig. 4d), while relative transcriptional efficiency of *dosR* and *dosS* are unchanged (Fig. 4c). This again points to a transcription-independent role for codon-biased translation in some cases. DosR protein levels were higher for ACG-biased mutants in hypoxia than WT, but lower in ACC- and ACT-biased mutants, possibly due to preferential paring of mcmo^5^U and cmo^5^U with G as in Thr^ACG^ (ref. 34). We also tracked DosR activity by measuring *hspX* expression. *hspX* is a prominent member of the *dosR* regulon, and its over-expression slows cell growth^35^. We found that *hspX* expression mirrors that of DosR levels (*R*^2^=0.75, *P*=3.8 × 10^−8^; Supplementary Fig. 9c), which implies that all four synonymous Thr codon mutants produced functional DosR protein that was translated in a timely manner but with differing efficiencies that reflect the previously observed codon bias effects (enhanced with ACG, reduced with ACC). The results of the codon re-engineering studies thus support the model of hypoxia-induced tRNA reprogramming linked to codon-biased translation of critical hypoxia response proteins.

Two clear conclusions emerge regarding the effect of *dosR* codon engineering on BCG survival under hypoxic conditions: (1) *dosS* and *dosR* are critical for BCG growth and survival under hypoxic conditions (Fig. 4e, Supplementary Fig. 9b) and (2) *dosR* codon substitution with the most strongly up- and downregulating codons (ACG and ACC, respectively; Fig. 3d) reduced BCG fitness during re-aeration (Fig. 4d). However, in spite of the clear codon-dependent changes in the expression of DosR, the master regulator of hypoxic dormancy, the survival phenotypes of the *dosR* codon-engineered mutants did not behave in a predictable manner. The ACC and ACG mutants had significantly reduced viability during re-aeration (Fig. 4e), which could reflect the consequences of significantly over- or under-expressing DosR during recovery from hypoxia. The lack of a strong correlation between DosR expression levels and hypoxic fitness points to influences from other systems and pathways in BCG, such as contributions from protein turnover kinetics, other stress response pathways (for example, enduring hypoxic response^36^), changes in protein secondary modifications (for example, phosphorylation of DosR^32^) and other signalling pathways, as well as transcriptional changes, all integrating in the final phenotype. For example, given that Majumdar *et al*.^37^ suggest that levels of DosR can decrease by as much as 7.5-fold in Mtb and still support HspX expression, we surmise that the lowered levels of DosR in ACC mutants was still sufficient to induce WT levels of *hspX* expression. These phenotypic complexities point to the need to better understand the integration of the many systems involved in mycobacterial persistence, while the *dosR* codon re-engineering results support the mechanistic importance of codon usage patterns in the expression of proteins critical to the hypoxic response in BCG.

## Discussion

**Figure 5:**
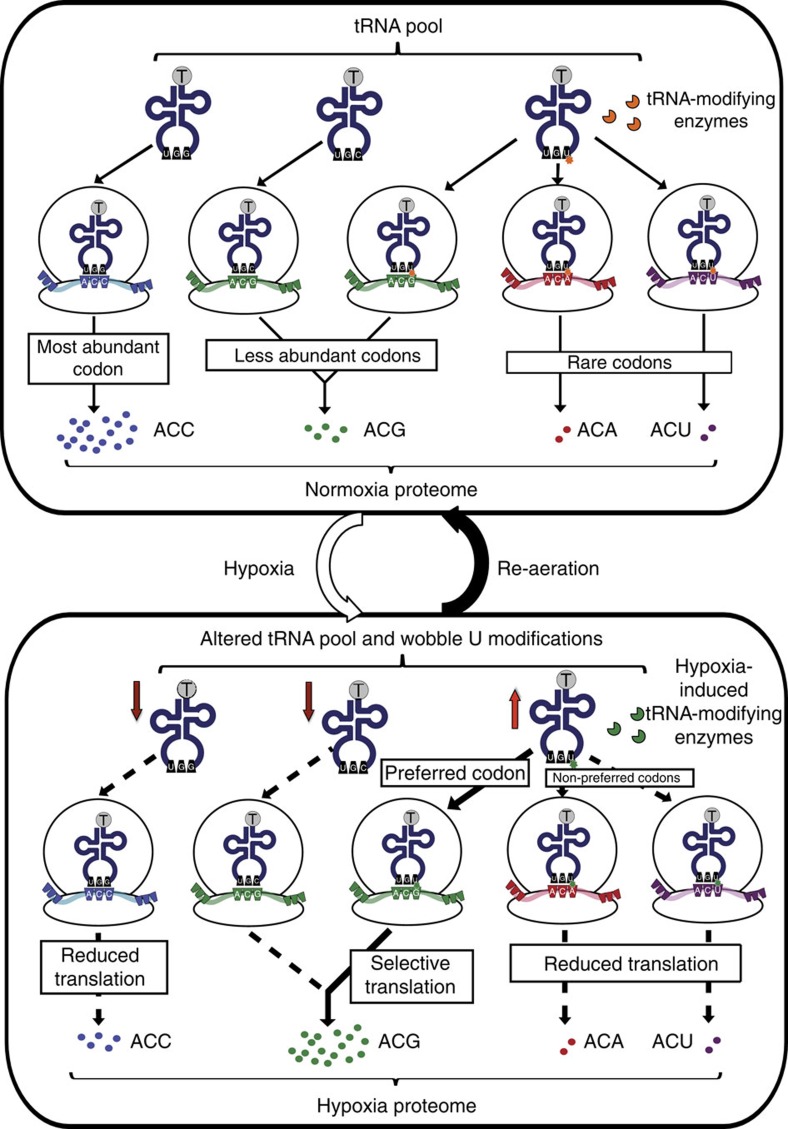
Proposed mechanism by which dynamic changes in the tRNA pool regulates selective translation of codon-biased transcripts and mycobacterial non-replicating persistence. Under normoxic conditions, translational selection is determined by the tRNA pool wherein highly expressed genes use a subset of optimal codons in accordance with their respective major isoacceptor tRNA levels (such as the codon ACC for Thr and its cognate tRNA^Thr^_GGU_ in BCG). Conversely, genes using less abundant or rare synonymous codons are expressed to a lesser extent. For instance, tRNA^Thr^_mo5UGU_ can read the codons ACG, ACA and ACU, diluting available tRNA^Thr^_UGU_ isoacceptors. Exposure to hypoxia alters the tRNA pool by both affecting both tRNA copy numbers and their modification status. Decreases in specific isoacceptors such as tRNA^Thr^_GGU_ and tRNA^Thr^_CGU_ funnel translation towards tRNA^Thr^_UGU_ decoding. Concurrent alternation of wobble mo^5^U to cmo^5^U shifts translational selection towards ACG-biased codons. The tRNA pool reverts upon re-aeration.

The totality of the results lead us to propose an integrated model for translational contributions to mycobacterial non-replicative persistence and to cellular stress response in general. Insights into the phenomenon of tRNA reprogramming and codon-biased translation, as well as the phenotypes of these synonymous Thr mutants, can be obtained by taking into account the interplay between codon usage, codon-tRNA pairings and changes in the tRNA^Thr^ pool (copy number, modification levels), as illustrated in Fig. 5. ACC—the optimal codon—and ACG are the two most abundant Thr codons in BCG and each is read by a single tRNA: tRNA^ThrGGU^ and tRNA^ThrUGU^, respectively. That these two tRNAs change copy number in opposite directions in early hypoxia (Fig. 2d, Supplementary Fig. 2f), accompanied by wobble reprogramming (Fig. 2e, Supplementary Fig. 2g) that amplifies ACG selectivity by tRNA^ThrUGU^, is consistent with hypoxia-induced upregulation of ACG-enriched proteins and downregulation of ACC-enriched proteins (Fig. 3). These conditions also explain the significantly enhanced expression of *dosR* in the hypoxic ACG-biased mutant (Fig. 4c,d). Moreover, our results with the ACG-biased mutant *dosR* (Fig. 4c,d) and published observations^38^ link overexpression of *dosR* with delayed aerobic growth. The poor fitness of the ACC mutant upon aerobic recovery can be explained by the fact that ACC is read by a single tRNA^ThrGGU^ isoacceptor that decreased significantly in copy number during HT (Supplementary Fig. 2f), which reduced the translation of ACC-biased *dosR* and slowed DosR accumulation (Fig. 4d, Supplementary Table 1). The delayed stress response encourages further growth in early hypoxia (H6 in Fig. 4e) but led to a loss of viability that was evident upon resuscitation (Fig. 4e). The fact that biased use of minor Thr codons, ACA and ACU, does not lead to dramatic effects on DosR expression or survival (Fig. 4d,e) may relate to the presence of two tRNA quasi-species that read each of these codons: tRNA^ThrGGU^ and tRNA^Thr(cmo5UGU)^ for ACU and tRNA^Thr(cmo5UGU)^ and tRNA^Thr(mo5UGU)^ for ACA (Supplementary Fig. 4). Opposing changes in copy number and modification in these pairs of tRNAs may negate any effect on expression of ACA- and ACU-biased *dosR* and thus survival (Figs 4e and 5).

These results suggest that BCG schedules transcripts for translation by reprogramming tRNAs to better read specific codons over their synonymous counterparts. Critical genes, such as *dosR*, use a specific set of codons that are transiently prioritized during stress to enhance their translation. Factoring in our previous observations in yeast^7^,^8^,^10^, a general model emerges in which the balance of codon usage and timing of tRNA modification changes in a cell is tuned to allow appropriate translation of survival and adaptation proteins under the right conditions (Fig. 5). It is important to point out here that, although as we have demonstrated in yeast^9^ and in the present studies, there is evidence for a disconnect between transcript levels and codon-biased translation, the model does not preclude contributions from transcriptional regulation. Indeed, this translational control system would cooperate with transcriptional mechanisms to provide a means to enhance the expression of critical proteins for which mRNA levels may not be optimal at the moment of a stress or for which transcript levels are abundant. With 3,000–8,000 mRNA molecules in a bacterial cell at any moment in time^39^,^40^ and mRNA copy numbers as low as 0.6–2 per gene, a translational control mechanism would compensate for non-optimal mRNA levels by scheduling the appropriate transcripts for translation under the pressure of a changing external environment. However, not all codon-biased transcripts are uniformly up- or downregulated, as illustrated by the fact that half of the proteins from ACG-enriched genes remain unchanged at any point in the hypoxia time course (Supplementary Table 2). This suggests that many factors contribute to stress-induced translational changes, such as mRNA abundance and other codon biases in the genes, with the observed stress-specific tRNA reprogramming and codon-biased translation enhancing translational efficiency as part of a larger network that regulates gene expression at all levels.

So what regulatory mechanisms lead to the tRNA reprogramming observed in mycobacteria in, for example, early hypoxia? There are clearly upstream transcriptional and translational events that are activated by hypoxia and precede the reprogramming and codon-biased upregulation of DosR, including an early increase in transcription of *dosR* (Supplementary Fig. 5e). The tRNA modification pattern on day 4 is clearly different from that of day 9 (Fig. 1b), which points to changes in gene expression that precede activation of the Dos regulon. Further downstream, the sensors DosS and DosT, which detect redox changes, hypoxia, nitric oxide and carbon monoxide, are histidine kinases that regulate DosR's activity as a transcription factor^32^. It is possible that these and other kinases affect earlier and later transcription and translation pathways, such as upregulation of the CmoA that converts wobble mo^5^U to cmo^5^U at day 9 of hypoxia in BCG. It is highly likely that the gradual changes in mycobacterial phenotype in response to hypoxic stress involve a cascade of regulatory events that includes stage-specific tRNA reprogramming and codon-biased translation. The codon usage, tRNA modification and proteomics data also support the idea that the tRNA modification reprogramming and codon-biased translation affect a larger network of proteins than just the DosR regulon in hypoxia and, more broadly, affect translation of other protein classes in other types of mycobacterial stress, such as nutrient deprivation. Of the 3,951 genes in BCG, 580 open reading frames possess codon usage patterns significantly deviating from the genome average (Supplementary Fig. 3), which presents a large number of genes that have the potential to be regulated in part by tRNA modification reprogramming during environmental changes.

## Methods

Chemicals and reagents, bacterial strains, media and growth conditions are described in Supplementary Methods.

### Identification and quantification of tRNA modifications

RNA extraction and purification is described in Supplementary Method 2. Purified BCG tRNA (0.5 μg per sample) was hydrolysed enzymatically as described elsewhere^22^. To prevent the formation of Tris-RNA adducts, HEPES buffer (pH 8.0) was used instead of Tris-HCl buffer (pH 8.0). Furthermore, all the enzymes were dialysed against HEPES buffer (pH 8.0) immediately before use. Reverse phase high performance liquid chromatography (HPLC) of the hydrolysed tRNA was performed as previously described^7^,^22^. Neutral loss scan, molecular feature extraction (MFE) and targeted ion fragmentation (targeted MS^n^) were used to identify 40 modifications in BCG tRNA. Neutral loss scan was performed on an Agilent 6460 LC-QQQ spectrometer with ESI Jetstream ionization, searching for compounds with loss of ribose (−136 *m/z*) or 2′-*O*-methyl-ribose (−146 *m/z*) upon fragmentation. High accuracy masses for molecular ions and CID fragments were obtained using either an Agilent 6520 LC-QTOF MS system with an ESI ionization or an LTQ Orbitrap XL MS system (Thermo-Scientific). Untargeted feature finding was performed using Molecular Feature Extraction (Agilent Workstation Qualitative Analysis vB05.06). Molecules reproducibly observed (by retention time, molecular mass, features of MS^2^ fragmentation) in all biological replicates at one time point were validated by comparisons with commercially available standards (see Supplementary Data 1), comparisons with theoretical molecular masses of ribonucleosides found in ChemSpider (http://www.chemspider.com/), Modomics (http://modomics.genesilico.pl/) and the RNA modifications database (http://mods.rna.albany.edu/), targeted MS^n^, isotopic envelope analysis and salt adduct analysis. Dynamic MRM on an Agilent 6460 LC-QQQ spectrometer was used to quantify these modifications^22^. Quantitative comparisons between biological replicates from various time points were made possible by correcting for biological variation in total tRNA quantities by dividing raw peak area for the ribonucleoside by the ultraviolet absorbance (in-line detector) peak areas for the four canonical ribonucleosides and normalizing spectra signals against that of the spiked internal standard ([^15^N]_5_-deoxyadenosine) to adjust for day-to-day fluctuation in MS sensitivity. All mass spectrometers were operated in positive ion mode. Relative quantification of tRNA modifications was performed as previously described^22^,^23^.

### Sequencing and quantification of tRNA-specific oligonucleotides

We adapted an liquid chromatography tandem mass spectrometry (LC-MS/MS)-based platform to map tRNA modifications, perform label-free absolute quantification of tRNA copy numbers and determine the extent of modification on those tRNA copies by combining the principles of bottom-up shotgun proteomics, amide-HILIC oligonucleotide liquid chromatography and response factor calibration outlined in previous studies^30^,^41^,^42^. However, the following alterations were made. The tRNA^Thr^ pool was characterized and quantified using Agilent 6520 QTOF and Agilent 6460 QQQ spectrometers coupled with an Agilent 1290 infinity LC system with online diode array for ultraviolet–visible spectrometry, operated in negative ion mode. BCG tRNA sequences were downloaded from the Genomic tRNA database (http://gtrnadb.ucsc.edu/) and RNase U2, T1 and A digestion products were predicted using Mongo Oligo Mass Calculator (http://mods.rna.albany.edu/masspec/Mongo-Oligo). Digestion products >6 nt in length were evaluated for sequence uniqueness (by BLASTn against genomic tRNA sequences) and RNAse U2 selected for further experiments as it generates unique products from the anticodon stem-loop of all three tRNAs for Thr^43^. We eliminated sequences with positional isomers that could be generated from RNAse U2 digests with or without missed cleavages, and identified a unique oligonucleotide tag for every RNA^Thr^. RNA and DNA oligomers with the same sequences (with the exception of U>T for DNA) were purchased and used to optimize LC and MS parameters for maximal chromatographic separation (by retention times), sequence coverage and signal strength of the fragments, unique transitions with high signal-to-noise ratios (S/N>10) and minimal source fragmentation.

RNase U2 (Thermo-Scientific) digestion of BCG tRNA was performed followed by the removal of 5′ and 3′ phosphates by bacterial alkaline phosphatase (Life Technologies)^44^. The reactions were performed in 10 mM ammonium acetate pH 7.0 at 37 °C for 4 h (2.5 h of U2 digest and 1.5 h of dephosphorylation) in the presence of deaminase inhibitors (0.5 μg ml^−1^ coformycin, 5 μg ml^−1^ tetrahydrouridine) and antioxidants (50 μM desferrioxamine, 50 μM butylated hydroxytoluene). The enzymes were dialysed against 10 mM ammonium acetate before use to prevent the formation of Tris-RNA adducts. The proteins were removed by filtration (Microcon YM-10), desalted by ZipTip_C18_ (Millipore) and concentrated by vacuum centrifuge. The extent of digestion and size of products were assessed by small Bioanalyzer chips (Agilent) and by MALDI-MS (Voyager DE, AB SCIEX) using 2,4,6-trihydroxyacetophenone (THAP) as matrix. The RNA fragments were reconstituted in 70% acetonitrile (v/v) for LC-MS/MS.

Amide-HILIC LC separation was performed on a TSK-gel Amide-80 column (2.0 mm ID × 150 mm, 3 μm particle size) using a binary solvent system consisting of 8 mM ammonium acetate in ultrapure water (solvent A) and acetonitrile (solvent B). HPLC was performed at a flow rate of 0.1 ml min^−1^. The gradient of solvent A was as follows: 0–2 min, held at 10% (v/v); 2–3 min, 10–15%; 3–5.5 min, 15–30%; 5.5–20.5 min, 30–60%; 20.5–25 min, 60–70%; 25–28 min, 70–10%. The HPLC column was maintained at 50 °C. QTOF mass spectrometer was operated at gas temperature 325 °C, gas flow 8 l min^−1^, nebulizer 30 p.s.i., Vcap 3,800 V, fragmentor 250 V, skimmer 1 65 V, octapole RF peak of 750. Targeted MS^2^ performed every 5 V from collision energies from 25 to 45 V and products scanned from *m/z* 100 to 1,500. QQQ mass spectrometer was operated at gas temperature 325 °C, gas flow 10 l min^−1^, nebulizer 32 p.s.i., sheath gas temperature 300 °C, sheath gas flow 11 l min^−1^, capillary 4,000 V, Vcharging 500. For MRM, MS1 acquisition was performed at wide resolution and MS^2^ acquisition at unit resolution. Dwell time per transition at 150 ms, fragmentor 130–160 V, collision energy 30–40 V and cell accelerator voltage 5–7 V.

As the positions of BCG tRNA modifications had not been mapped, we generated a list of potential modified oligomers by taking into account the 40 modifications identified in BCG tRNA (Fig. 1; Supplementary Data 1), predicted modifications by tRNAmod (http://crdd.osdd.net/raghava/trnamod/) and comparisons with tRNA sequences catalogued in Modomics (http://modomics.genesilico.pl/). The exact masses of these potential modified oligomers and their c1 and y1 ion fragments were calculated. Selected reaction monitoring (SRM) worklists were created to screen the tRNA pool of Log, H18 and R6 cells for oligomers with these predicted c1 and y1 transitions. Selected reaction monitoring screen for c1 and y1 transitions were performed separately and in technical duplicate on the LC-QQQ. Oligomers with matching the retention times for their predicted c1 and y1 transitions were sequenced by targeted MS^2^ on the LC-QTOF at collision energies of 0, 15, 30 and 45 V. Fragment analysis, aided by SOS^45^ and RoboOligo^46^, enabled us to map the exact location of each modification based on their a-B, c, w and y ions. The analysis of free bases and internal fragments (mainly matching ion fragments with w type cuts at the 3′ end or a-B type cut from the 5′ end, generating a pNpf or NpNf fragment) further validated our structural assignments^47^. The two strongest signals (Supplementary Fig. 2, Supplementary Data 3) were selected to quantify and identify each modified oligomer by MRM in the same run. Response factors for RNA oligomers against DNA oligomers of the same sequence were computed by external calibration (Supplementary Fig. 2). With the assumption that these response factors are applicable to modified oligomers of the same length and sequence, we quantified the amounts of each tRNA species by the ratios of peak areas between the quantifier transitions of the modified RNA fragment to 1 pmol of spiked DNA oligomers of the same length and sequence. Signal processing and data reduction procedures for oligonucleotide mapping, scoring of *de novo* sequences and identity validation through the use of synthetic oligonucleotides are described in Supplementary Methods.

### iTRAQ labelling and peptide fractionation

Protein extraction and processing is described in Supplementary Methods. Aliquots of digested protein (from 50 μg of total protein) were split in three as technical replicates and labelled with 8-plex iTRAQ reagents according to the manufacturer's instructions. To avoid bias from any one tag during analysis, labels for samples from each time point were randomized and unblinded post-analysis. After iTRAQ labelling, the samples were desalted with Sep-Pak Plus C18 cartridges (Waters), dried by vacuum centrifugation and reconstituted in IPG buffer (Agilent) without glycerol. Isoelectric focusing was performed from pH 3 to 10 over 24 wells on an Agilent 3100 OFFGEL fractionator according to the manufacturer's protocol (OG24PE00). All the 24 fractions were collected and analysed by nano-LC-MS/MS.

### LC-MS/MS analysis of the BCG proteome

iTRAQ proteomics experiments were performed on an Agilent 1200 nano-LC-Chip/MS interfaced to an Agilent 6510 QTOF LC/MS. The LC system consisted of a capillary pump for sample loading, a nanoflow pump and a thermostated microwell-plate autosampler. The HPLC-Chip configuration consisted of a 160 nl enrichment column and a 150 mm × 75 μm analytical column (G4240-62001 Zorbax 300SB-C18). Mobile phases used were: 0.1% formic acid in water (solvent A) and 0.1% formic acid in acetonitrile (solvent B). A 120 min long gradient LC separation was used with 10 min for column wash and equilibration between runs. The samples were loaded into the enrichment column at 1% (v/v) B at flow rates of 3 μl min^−1^. On the nano-flow pump, the gradient of solvent B was as follows: 0–1 min, held at 1% (v/v), flow rate from 0.4 to 0.2 μl min^−1^; 1–101 min, 1–45%, flow rate held at 0.2 μl min^−1^; 101–121 min, 45–75%, flow rate held at 0.2 μl min^−1^; 121–122 min, 75–98%, flow rate from 0.2 to 0.4 μl min^−1^; 122–126 min, held at 98%, flow rate held at 0.4 μl min^−1^; 126–127 min, 98–1%, flow rate held at 0.4 μl min^−1^; 127–130 min, held at 1%, flow rate held at 0.4 μl min^−1^. LC-QTOF was operated at high resolution (4 GHz) in positive ion mode with the following source conditions: gas temperature 325 °C, drying gas 5 l min^−1^, fragmentor 225 V. Capillary voltage was adjusted between 1,500 and 2,100 V manually to achieve a steady spray. The data were acquired from 200 to 1,700 *m*/*z* with an acquisition rate of 4 spectra s^−1^ in MS mode and from 50 to 2,200 *m*/*z* with an acquisition rate of 2 spectra s^−1^ in MS/MS mode.

LC/MS data were extracted and evaluated using the MFE algorithm in MassHunter Qualitative Analysis software (B04.00). Test injections (three to four) from each fraction of the first technical replicate were made to optimize injection volumes for the second and third biological replicates for maximal extracted molecules with peptide-like features. For each fraction, the MFE list of molecular ions was exported and used to exclude the acquisition of spectra from these ions in subsequent runs. As such, every fraction from each technical replicate was run twice, first without and later with the exclusion list. Data from MassHunter Qualitative Analysis was exported to Mass Profiler Professional (version B02.02) for analysis of technical reproducibility. This process was repeated for all three biological replicates. Mass spectra were processed using Spectra Mill (Agilent; v B.04.00.127), X!Tandem (The GPM, thegpm.org; version CYCLONE (2010.12.01.1)) and Scaffold (version Scaffold_4.3.0, Proteome Software Inc.) as detailed in Supplementary Method 3.

### Strain construction

The strains used in this study are derivatives of BCG str. Pasteur 1173P2 and are listed in Supplementary Table 3. *dosSR* knockout and complementation are constructed by published methods^48^,^49^,^50^,^51^. Briefly, primers *dosR*_*hr*_FR, *dosS*_*hr*_FR and *dosRreg*FR (sequences in Supplementary Table 3) were used to amplify the *dosR* 3′ flanking region, *dosS* 5′ flanking region and *dosSR* promoter regions, respectively (Supplementary Fig. 9a). The promoter regions encompass all identified promoter and transcription factor binding elements identified^52^,^53^,^54^. These regions were subcloned into TOPO vector by TA cloning (TOPO TA cloning kit, Life Technologies), propagated in TOP10 chemically competent *E. coli*, and sequenced to select for vectors that correctly amplified the PCR fragment. Inserts were excised by the appropriate restriction enzymes and ligated into pYUB854 (for *dosS*_*hr*_FR and *dosRreg*FR inserts) or pMV306 (for *dosR*_*reg*_FR insert) vectors using the DNA Ligation Kit Mighty Mix (Takara) according to the manufacturer's instructions. Transfection of WT BCG with pYUB854 containing the *dosR*_*hr*_FR and *dosS*_*hr*_FR inserts generated the Δ*dosSR* strain. Δ*dosSR* was in turn complemented with pMV306 containing the *dosR*_*reg*_FR insert and one of five possible *dosSR* constructs (synthesized by g-blocks (IDT) and altered by site-directed mutagenesis (Genescript). This generated the Δ*dosSR*::*dosSR(WT)*, Δ*dosSR*::*dosSR(ACA)*, Δ*dosSR*::*dosSR(ACC)*, Δ*dosSR*::*dosSR(ACG)* and Δ*dosSR*::*dosSR(ACT)* strains. Deletion at the correct locus was verified by hygromycin resistance, gel electrophoresis of PCR products and by quantitative PCR (qPCR). Successful complementation was determined by kanamycin resistance, restriction mapping, sequencing and qPCR. The stability of the secondary structures of the re-engineered dosR constructs were analysed by mfold (http://mfold.rna.albany.edu). All sequences possessed identical 3′ and 5′ structures (no changes were made in the flanking dosS and BCG_3157c sequences) and had only modest changes to local dosR secondary structures (ΔG0 were stable at 28–32 kcal mol^−1^ for the top five structures for all dosR sequences—wild type and mutants).

### Reverse transcription–qPCR

Quantification of targeted mRNA sequences was performed as described in ref. 55, using primers in Supplementary Table 3. *sigA* served as internal loading control.

### Targeted quantification of DosS and DosR by Skyline and AQUA

Skyline, an open source software application (http://proteome.gs.washington.edu/software/skyline), was used to build MRM-MS experiments for the targeted quantification of DosS and DosR protein. The standard protocol, as described in ref. 56 was used; wherein DosS and DosR sequences were pasted unto the Skyline document and search against a BCG background database built from SwissProt.BCG.Pasteur.1173P2.fasta (archived on the CHORUS database, Project ID 1107). Automated picking of precursor peptides and transitions was used with filters set to select for singly charged, long (y3 and greater) y-ions with no *m/z* overlaps with b-ions. The method containing all valid *in silico* predicted MRM transitions was exported and used on an Agilent 6460 QQQ spectrometers coupled with an Agilent 1290 infinity LC system with online diode array for ultraviolet–visible spectrometry, operated in positive ion mode. Proteins were extracted from samples of wild-type and mutant BCG at various stages of the hypoxia-aerobic resuscitation time course using the method described above and separated by reverse phase HPLC using a Zorbax 300SB-C18 (4.6 × 12.5 mm, 5 μm; Agilent) guard column for desalting before introduction to a Zorbax RRHD 300SB-C18 analytical column (2.1 × 100 mm, 1.8 μm; Agilent). Reverse phase separation was performed with the following gradient of water and acetonitrile acidified with 0.1% (v/v) formic acid with a gradient of solvent B was as follows: 0–0.5 min, held at 1% (v/v), flow rate from 0.3–0.1 ml min^−1^; 0.5–60 min, 1–50%, flow rate held at 0.1 ml min^−1^; 60–61 min, 50–2%, flow rate held at 0.1 ml min^−1^; 61–63 min, held at 2%, flow rate from 0.1 to 0.3 ml min^−1^; 63–64 min, 2–1%, flow rate held at 0.3 ml min^−1^. Source conditions: gas temperature 325 °C, gas flow 10 l min^−1^, nebulizer 32 p.s.i., sheath gas temperature 300 °C, sheath gas flow 11 l min^−1^, capillary 2,000 V, Vcharging 500. Columns were incubated at 40 °C.

Results were imported back into Skyline for method refinement. iTRAQ proteomics data (from this study) was imported as a spectral libraries and used to validate ion ratios in the absence of internal standards (Supplementary Fig. 10a–h). The top two precursor peptides by peak area and number of transitions (minimum two) were selected as qualification and quantification ions and Agilent Automated MRM Method Optimizer for Peptides used to optimize collision energies and fragmentation voltages for their MRM transitions. Optimized parameters for quantifiers and qualifier transitions (Supplementary Table 3) were: QDPLSGLTDQER—collision energy 14.1 V, fragmentor 130 V; DIATELLSGTEPATVFR—collision energy 19.9 V, fragmentor 125 V; TLLGLLSEGLTNK—collision energy 19.1 V, fragmentor 130 V; TIPVAGAVLR—collision energy 9.9 V, fragmentor 140 V. Cell accelerator voltage was kept constant at 7 V.

Targeted AQUA analysis^57^ was performed by using a labelled peptide stock solution—prepared by dissolving isotopically labelled versions of the two target peptides (QDPLSGLTDQER* and DIATELLSGTEPATVFR*, where R* is U-^13^C_6_, ^15^N_4_ Arg with a Δ*m*/*z* of 10) in 0.1% (v/v) formic acid and 1% (v/v) acetonitrile in water to a final concentration of 50 fmol μl^−1^—to dissolve the vacuum-dried protein digest of each sample. The samples were analysed using the LC-QQQ using optimized parameters. To calculate calibration curves for quantification, protein digests from Δ*dosSR* were spiked with the indicated amounts of unlabelled quantifier peptides and their peak area ratios relative to 100 fmol of their corresponding AQUA peptides plotted (Supplementary Fig. 10i–l). The quantity of each peptide (and corresponding protein) relative to Log was determined.

### Analysis of codon usage within operons

A total 2,322 annotated operons in BCG were downloaded from Door^2^ (http://csbl.bmb.uga.edu/DOOR/). Nine hundred and sixty-five proteins that can be reproducibly quantified form the iTRAQ experiment were mapped onto their respective operons using custom Matlab (R2015a) scripts while picking out operons with more than one gene member. We then manually validated whether these operons produced polycistronic mRNA by comparison with RNAseq reads (NCBI Gene Expression Omnibus study GSE66883). Pair-wise comparisons of protein expression of gene neighbours throughout the hypoxia time course were made and their *R*^2^ coefficient of regression statistic calculated. Gene pairs with *R*^2^ greater than 0.4 are set as correlated while those with R^2^ less than −0.4 are set as anti-correlated pairs. gene-specific codon counting^10^ was used to quantify the codon usage differences between partners within correlated and anti-correlated pairs. The difference in codon usage frequencies were then summed and visualized (Supplementary Fig. 8). A codon is set to be over-represented in genes with anti-correlated protein abundance changes if its cumulative frequency is more than twice its cumulative frequency in correlated pairs and vice versa. The strength of the association between ACG codon usage and anti-correlated protein expression between gene pairs is then assessed using relative risk by assuming that codon usage of a gene is independent of protein expression correlation between operonic gene neighbour pairs (Supplementary Fig. 8). Gene pairs in which one partner was enriched with Thr^ACC^ and the other with Thr^ACG^ are 4.6 times (confidence interval: 2.7–8.1, *P*<0.0001) more likely to be anti-correlated in protein expression.

### Statistical analysis

All the experiments involved triplicate cultures, repeated at least four independent times. The data from mass spectrometric measurements and gene expression were deflated as fold changes against Log values. Logarithmic transformations were used for data sets with skewed or wide distributions and indicated in their respective figures legends. The data from bacterial growth were curve-fitted to an order 5 polynominal curve using Excel's LINEST function (Microsoft). Comparisons between the two samples were made using the appropriate two-tailed *t*-tests after equality of variances were tested using *F*-tests. Comparisons between multiple samples were determined using one-way or two-way analysis of variance when comparing one or two factors, respectively. Bonferroni's or Dunnett's Multiple Comparison or Tukey's HSD *post hoc* tests were used where it best reflects how sample means were compared and stated in their respective figure legends. These statistical tests were performed using Prism 5 (Graphpad). Unless otherwise stated, all the data are represented as arithmetic means±s.e. To aid interpretation on statistical significances, *P*<0.05, *P*<0.01 and *P*<0.001 are denoted as *, ** and ***, respectively. Multivariate statistical analysis of the meta-data is described in Supplementary Methods.

### Data availability

All proteomics data from this study are available through the CHORUS mass spectrometric data repository at (https://chorusproject.org/; Project ID 1107). Supporting RNA-Seq data is available from the Gene Expression Omnibus data repository with accession code GSE66883. The data that support the findings of this study are available from the corresponding author upon request.

## Additional information

**How to cite this article:** Chionh, Y. H. *et al*. tRNA-mediated codon-biased translation in mycobacterial hypoxic persistence. *Nat. Commun.* **7,** 13302 doi: 10.1038/ncomms13302 (2016).

**Publisher's note:** Springer Nature remains neutral with regard to jurisdictional claims in published maps and institutional affiliations.

## Supplementary Material

Supplementary Information Supplementary Figures 1-10, Supplementary Tables 1-3, Supplementary Methods and Supplementary References

Supplementary Data 1 Modified ribonucleosides in *Mycobacterium bovis* BCG tRNA

Supplementary Data 2 Changes in tRNA modifications in BCG during hypoxia and subsequent reaeration

Supplementary Data 3 Targeted Mapping of Modified Ribonucleosides in tRNA^Thr^ Isoacceptors

Supplementary Data 4 Changes in protein abundance as log_2_(fold-change) against Log-growing cells
