# Supplementary material for: tRNA-mediated codon-biased translation in mycobacterial hypoxic persistence
Source: Nat Commun. 2016 Nov 11;7:13302. doi: 10.1038/ncomms13302 (PMC5114619; doi:10.1038/ncomms13302)
Supplement: Supplementary Information — Supplementary Figures 1-10, Supplementary Tables 1-3, Supplementary Methods and Supplementary References [file ncomms13302-s1.pdf]

Supplementary Figure 1

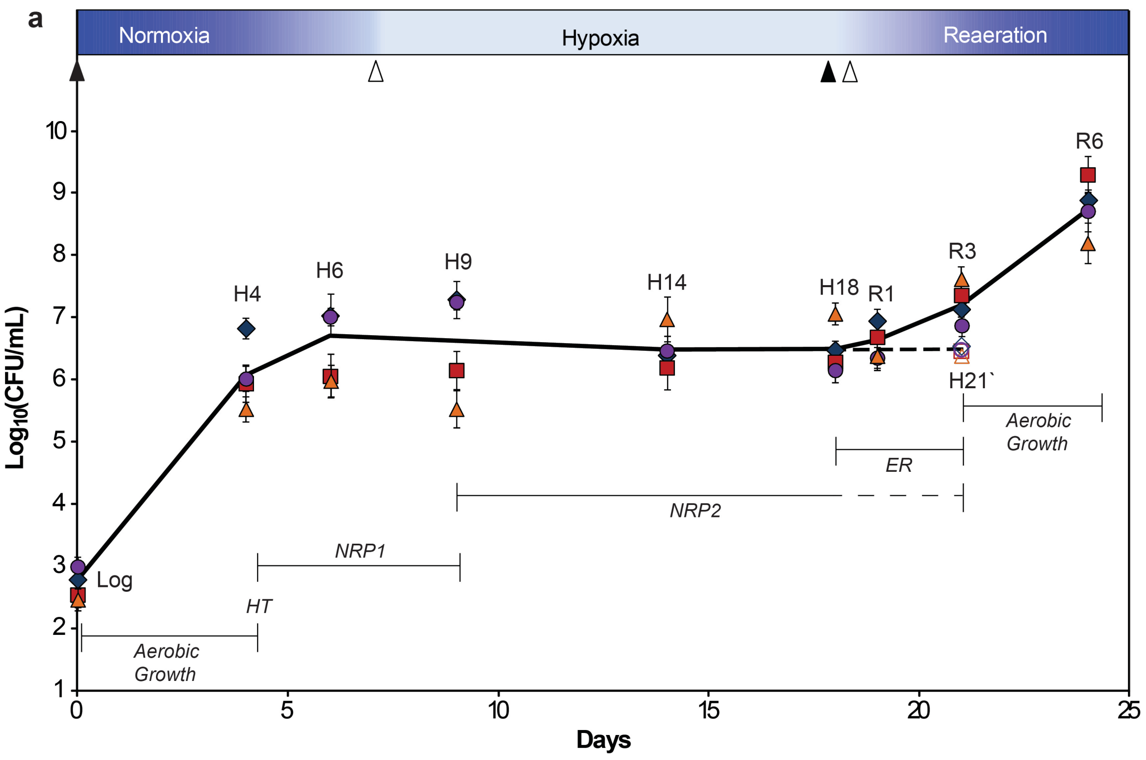

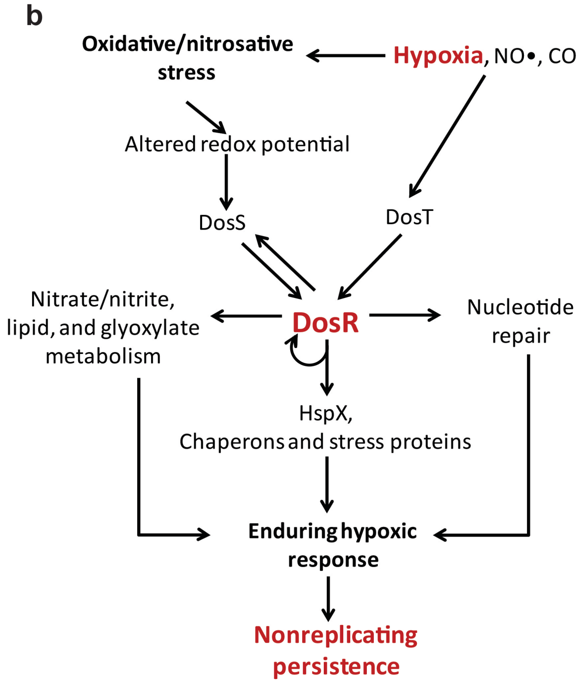

Supplementary Figure 1. Hypoxia-induced non-replicating persistence in *M. bovis* BCG. (**a**) Growth of BCG in the Wayne-like culture system adapted by Ravindran *et al.*[1] Symbols each represent the means for ≥3 biological replicate cultures in the same study; error bars: relative error of means. Methylene blue (MB) decolorization was used to gauge O_2 levels in cultures. Blue-shaded top panel: Intensity plot of MB decolorization upon reduction, with leucomethylene blue tracked at A_{665} and correlated to color intensity between blue (100% - initial A_{665}) and white (0% - A_{665} = 0). Flasks were sealed at day 1 and opened at day 18 (black arrows), with complete MB decolorization observed at day 7 and recolorization at 18.5 (white arrows). To determine if cultures remained non-replicative and viable if flasks remained sealed, one flask from each batch remained sealed for another 3 days to day 21 (denoted H21`). Time points noted as Log (day 0), H4 (hypoxia day 4), H6 (day 6), H9 (day 9), H14 (day 14), H21` (day 21- dashed lines), R3 (reaerated/resuscitated at day 21) and R6 (day 24). Stage of growth noted in *italics:* aerobic growth, *HT* (hypoxic transition), *NRP2* (non-replicating persistence stage 1), *NRP2* (non-replicating persistence stage 2), *ER* (early resuscitation). No statistically significant changes in CFU/mL were detected for H4-H18, R1, R3 and H21` samples by one-way ANOVA with Tukey's HSD. (**b**) Model for the regulation of hypoxia-induced non-replicating persistence by DosR. Under hypoxia, sensor histidine kinases DosS and DosT activate DosR, which upregulates the *dos* regulon (*dosR*, *dosS* are members), leading to changes in cellular metabolism, protein homeostasis and nucleotide repair. A subsequent enduring hypoxic response is induced, which enables the cell to enter non-replicating persistence[2-6].

Supplementary Figure 2

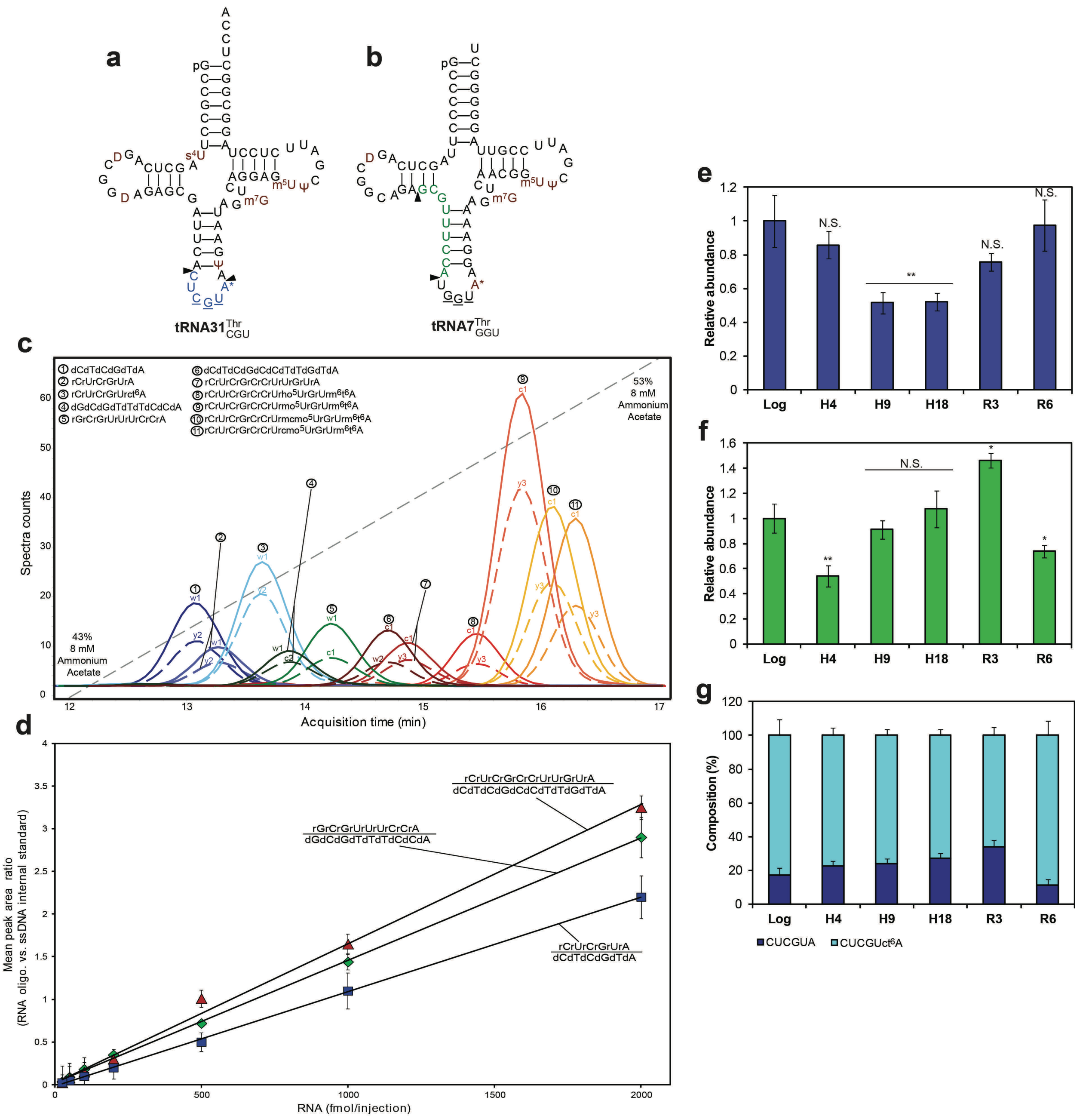

Supplementary Figure 2. Hypoxia induced changes in tRNA$^{Thr(\underline{C}GU)}$ and tRNA$^{Thr(\underline{G}GU)}$. Unique identifier oligos generated from RNase U2 digests for (**a**) tRNA31$^{Thr(\underline{C}GU)}$ and (**b**) tRNA31$^{Thr(\underline{G}GU)}$ (arrows) for LC-MS modification mapping and copy number analysis. Identifier oligos for tRNA46$^{Thr(\underline{U}GU)}$ are shown in **Fig. 2a**. (**c**) Oligos (1-11, precursor ion) for members of the tRNA^{Thr} pool were quantified by label-free absolute quantification. Panel shows composite extracted ion chromatograms of MRM transitions (peaks labeled with identity of product ion) used to quantify unique RNase U2 fragments that define each tRNA^{Thr}. Dashed line shows HPLC elution gradient. (**d**) Calibration curves for the response of RNA oligos against 1 pmol of their DNA counterparts and across 25-2000 fmol RNA. Mean peak area ratios were fitted to linear equations, with correlation coefficients (R^2) >0.995 for all 3 curves. Instrument response reproducibility was studied by triplicate injections, with RSD <11 % for all concentrations; response factors were calculated from the slope of these curves. MRM transitions used are found in **Supplementary Data 1**. (**e, f**) Fold-change in copy number of (**e**) tRNA$^{Thr(\underline{C}GU)}$ and (**f**) tRNA$^{Thr(\underline{G}GU)}$ at H4, H9, H18, R3 and R6 vs. Log. A total of 237 (±64) copies of tRNA$^{Thr(\underline{C}GU)}$ and 1245 (±290) copies of tRNA$^{Thr(\underline{G}GU)}$ were present per CFU at Log, as determined by SRM. Data represent mean ± SEM; n = 4. Statistical analysis by one-way ANOVA with Dunnett's test vs Log: NS, not significant; $p< 0.05$, $p < 0.01$ and $p < 0.001$ are denoted as *, ** and ***, respectively). (**g**) Composition of the tRNA$^{Thr(\underline{C}GU)}$ pool in terms of its A37 modification variants at indicated time points expressed as percentage of sum total. This illustrates the constancy of the ct^{6}A modification at position 37 as a control for changes in the wobble modifications.

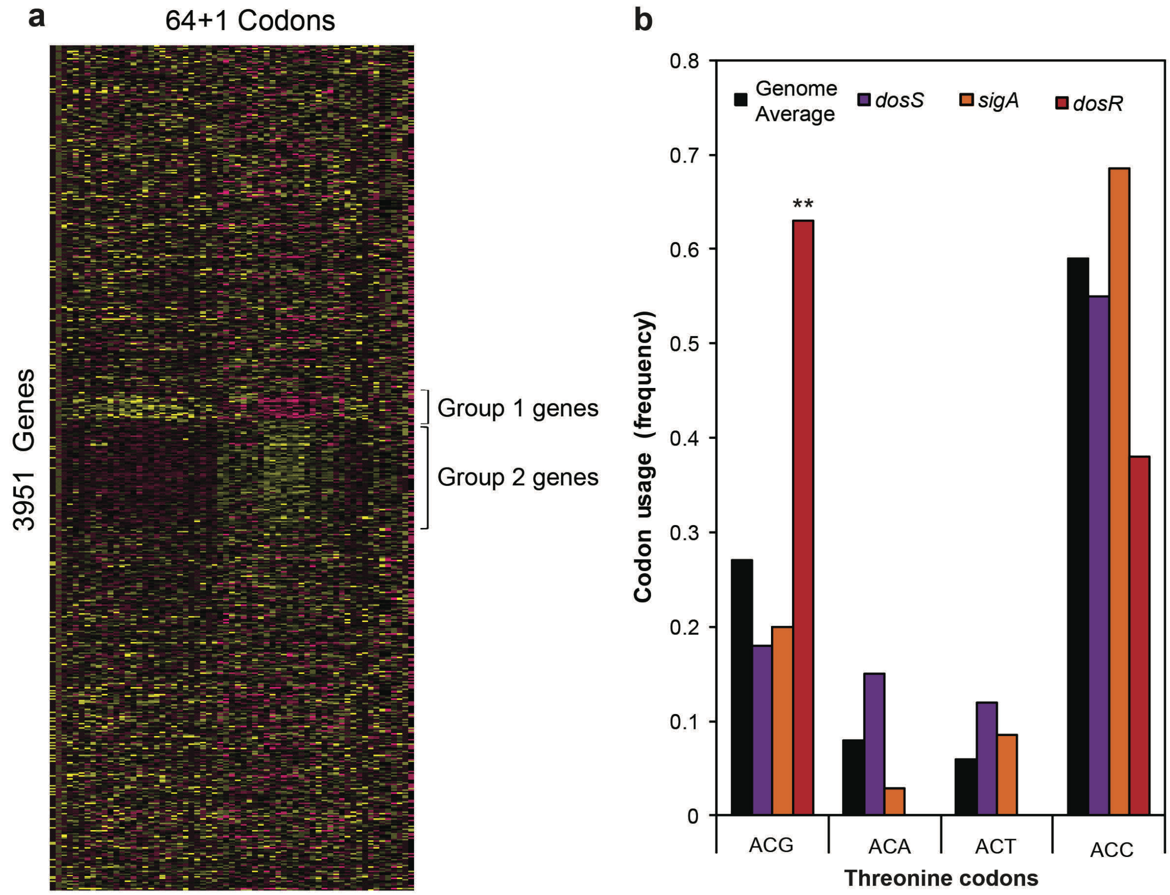

Supplementary Figure 3. Codon usage patterns in the BCG genome. (**a**) Heat map of codon usage patterns across the BCG genome. The gene-specific codon counting (GSCC) algorithm was used to analyze all 3951 protein-coding genes in BCG for all codons choices (including alternate start '+1' and stop codons)[7]. Visualization by heat map of over-used (yellow) and under-used (purple) codons revealed 580 open reading frames that possessed codon usages significantly deviating from the genome average (Cohen's d > 0.5). **Group 1** genes over-use Ala^{GCT}, Asn^{AAT}, Asp^{GAT}, Cys^{TGT}, Gly^{GGT}, His^{CAT}, Ile^{ATA}, Ile^{ATT}, Leu^{TTA}, Leu^{TTG}, Phe^{TTT}, Pro^{CCT}, Thr^{ACG}, Tyr^{TAT}, and Val^{GTT}, and under-use Ala^{GCC}, Arg^{CGC}, Asn^{AAC}, Asp^{GAC}, Cys^{TGC}, Gly^{GGC}, His^{CAC}, Ile^{ATC}, Leu^{CTC}, Leu^{CTG}, Phe^{TTC}, Pro^{CCC}, Thr^{ACC}, Tyr^{TAC}, and Val^{GTC}. **Group 2** genes over-use Ala^{GCC}, Arg^{CGC}, Asn^{AAC}, Asp^{GAC}, Gly^{GGC}, His^{CAC}, Ile^{ATC}, Leu^{CTG}, Phe^{TTC}, and Thr^{ACC}, and under-use Ala^{GCA}, Ala^{GCT}, Arg^{CGA}, Asn^{AAT}, Asp^{GAT}, Gly^{GGT}, His^{CAT}, Ile^{ATA}, Ile^{ATT}, Leu^{TTG}, Leu^{CTT}, Phe^{TTT}, Pro^{CCA}, Pro^{CCT}, Ser^{AGT}, Thr^{ACA}, Thr^{ACG}, Thr^{ACT}, Val^{GTA} and Val^{GTT}. Twelve of these codon choices feature as determinants of protein up- or down-regulation during hypoxic and normoxic shifts (**Fig. 3d, Supplementary Fig. 7**). (**b**) Usage frequencies of synonymous Thr codons, ACG, ACA, ACT and ACC, across all protein-coding genes in the genome (genome average) and in genes *dosS*, *sigA* and *dosR*.

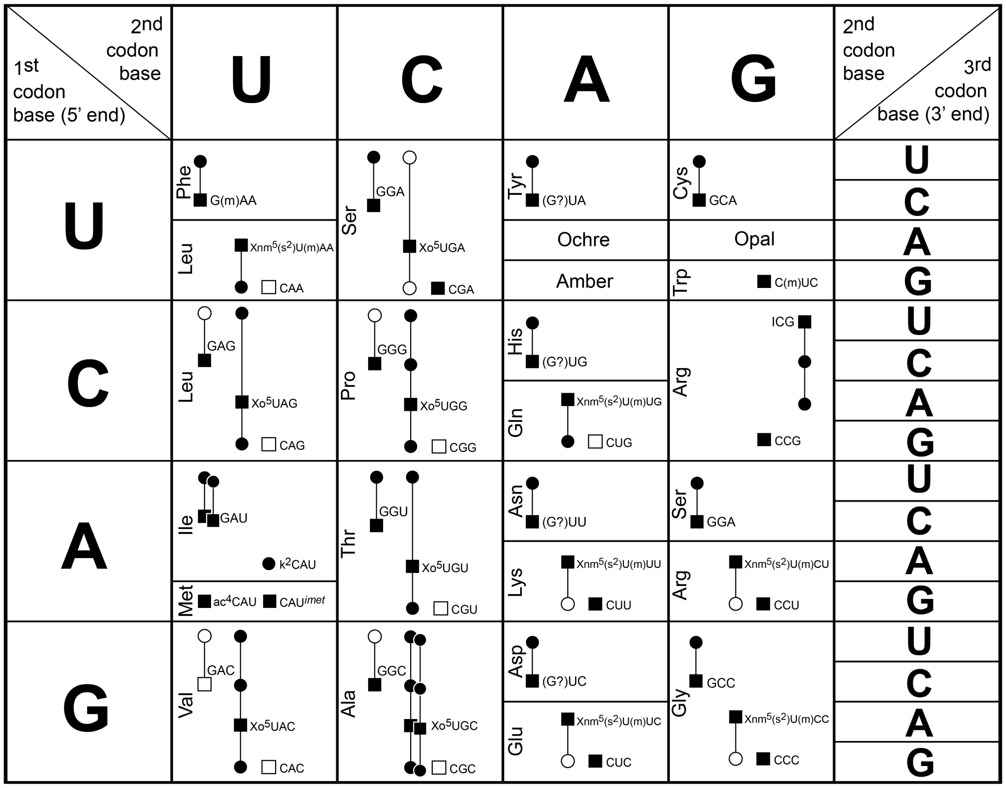

Supplementary Figure 4. Presumptive genetic decoding in BCG. In this coding chart, which is based on the work of Bjork *et al.*[8], symbols connected with lines indicate codons that are read by the same tRNA. Stacked symbols in AUH and GCN boxes represent isodecoder pairs for tRNA$^{Ile(\underline{G}AU)}$ and tRNA$^{Ala(\underline{U}CC)}$, respectively. Squares denote cognate Watson-Crick base pairings while circles denote non-cognate pairings. Filled symbols denote productive pairings between codon and anticodons that had been validated experimentally in prokaryotes[9-14]. Codon-anticodon pairings assigned based on wobble rules of Crick[15], and observations of Yokoyama and Nishimura[16], Agris *et al.*[17], Grosjean *et al.*[18] and Roth[19]. Xnm^5(s^2)U$_m$: 5-iminomethyl-U$_{34}$ family of hypermodified uridines in which mnm^{5}U, mnm^{5}s^{2}U, cmnm^{5}s^{2}U and cmnm^{5}U$_m$ had been found in BCG tRNA. Xo^{5}U: 5-oxyU$_{34}$ family of hypermodified uridines in which ho^{5}U, mo^{5}U, cmo^{5}U and mcmo^{5}U had been found in BCG tRNA. (G?): Queuosine (G$_{34}$) family of hypermodified guanosines; however, no members had been detected in this study. Full names, chemical structures and LC-MS/MS characteristics of all modifications can be found in **Supplementary Data 1**. Amber, Ochre and Opal are stop codons. Only F-met^{ATG}, the canonical start codon, is featured in this scheme.

Supplementary Figure 5

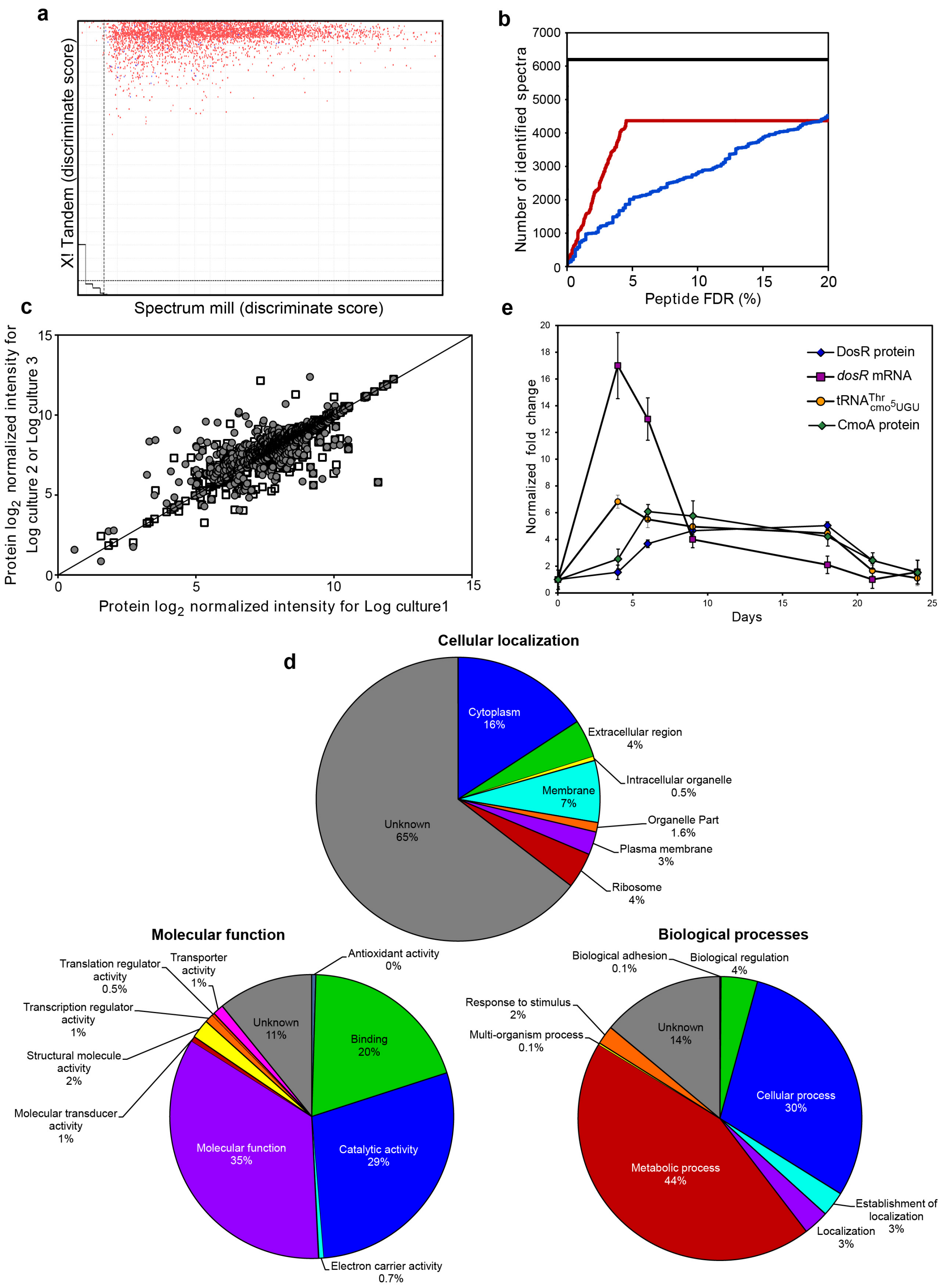

Supplementary Figure 5. Proteomic analysis of BCG entering and emerging from hypoxia-induced non-replicative persistence. (**a**) Scatterplot of spectrum/peptide matches between X!Tandem and Spectrum Mill. The x- and y-axes are the search engines' discriminant scores determined by Scaffold. Vertical and horizontal dashed lines define the 95% peptide probability filter. Correct peptide-to-protein assignments are noted in red; incorrect in blue. (**b**) Peptide receiver operator curve (ROC) plot for the selection of global peptide false discovery rate (FDR). Sensitivity is defined as the number of correctly identified spectra (y-axis; true positives); fall-out is defined as the FDR (x-axis, false positives). Blue, red and black traces are the ROCs for Spectrum Mill, X!Tandem and combined scores, respectively. At 4.9% FDR (plateau in X!Tandem scores), 2455 proteins were identified. (**c**) Scatter plot for intensities of iTRAQ labels (randomized for each biological replicate) for all proteins in Log samples. The x-axis plots the quantitative values for the first biological replicate; y-axis plots the quantitative values for the second (□) and third (●) biological replicate. The 965 most quantifiable proteins fall within the 95% confidence interval of the optimal 45° reporter ion correlation line that represents a 1:1 ratio between the selected quantitative samples. (**d**) Gene ontology analysis of the 965 most quantifiable proteins. Classification of the protein set was performed according to the 1st level gene ontology terms: "Cellular component", "Molecular function" and "Biological process". Pie charts show the breakdown according to 2nd level classifications. Molecular function in (**d**) could be further parsed into categories for binding, electron carrier activity, enzyme regulator activity, guanyl-nucleotide exchange factor activity, metallochaperone activity, nutrient reservoir activity, receptor activity, receptor regulator activity. (**e**) Changes in the abundances of DosR protein, *dosR* mRNA, tRNA$^{Thr(\underline{cmo5U}GU)}$ and putative CmoA protein across the hypoxia time course (**Supplementary Fig. 1a**). All abundances are normalized to their values at day 0 (Log) as fold-changes.

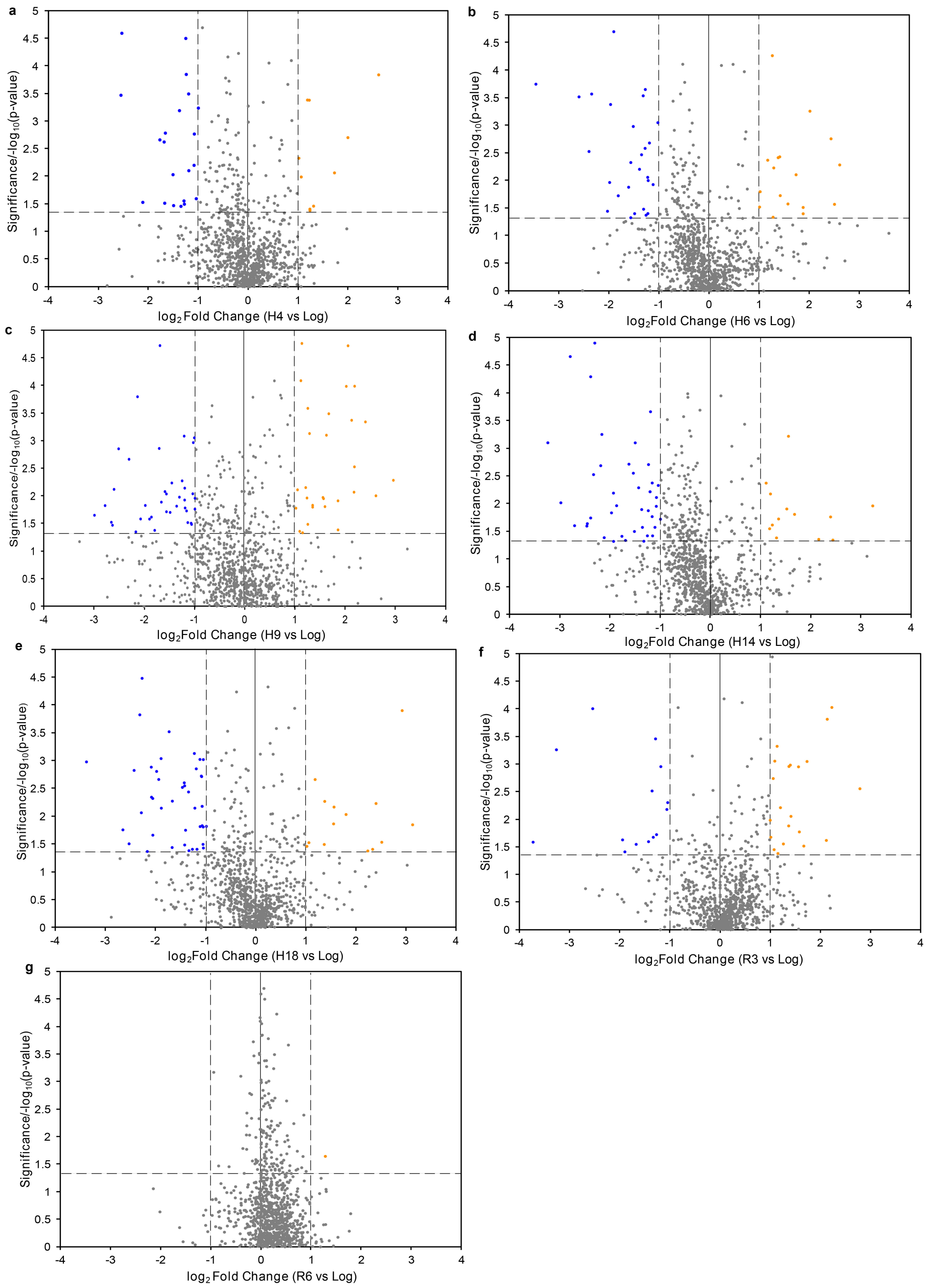

Supplementary Figure 6. Identity-agnostic selection of proteins that changed significantly in abundance during the entry and exit from hypoxia-induced bacteriostasis. Volcano plots help to visualize the proteins that were significantly up- or down-regulated at each hypoxia time point compared to Log conditions: (**a**) H4, (**b**) H6, (**c**) H9, (**d**) H14, (**e**) H18, (**f**) R3, and (**g**) R6. The X-axis indicates the differential protein expression profiles, plotting the fold-induction ratios on a $\log_2$ scale. The Y-axis indicates the statistical significance of the difference in expression (p-value from a t-test) in a $\log_{10}$ scale. Proteins up-regulated >2-fold with $p < 0.05$ are shown in blue; proteins down-regulated >2-fold with $p < 0.05$ are shown in orange. Fold-changes of the 965 proteins analyzed are presented in **Supplementary Data 4**.

Supplementary Figure 7

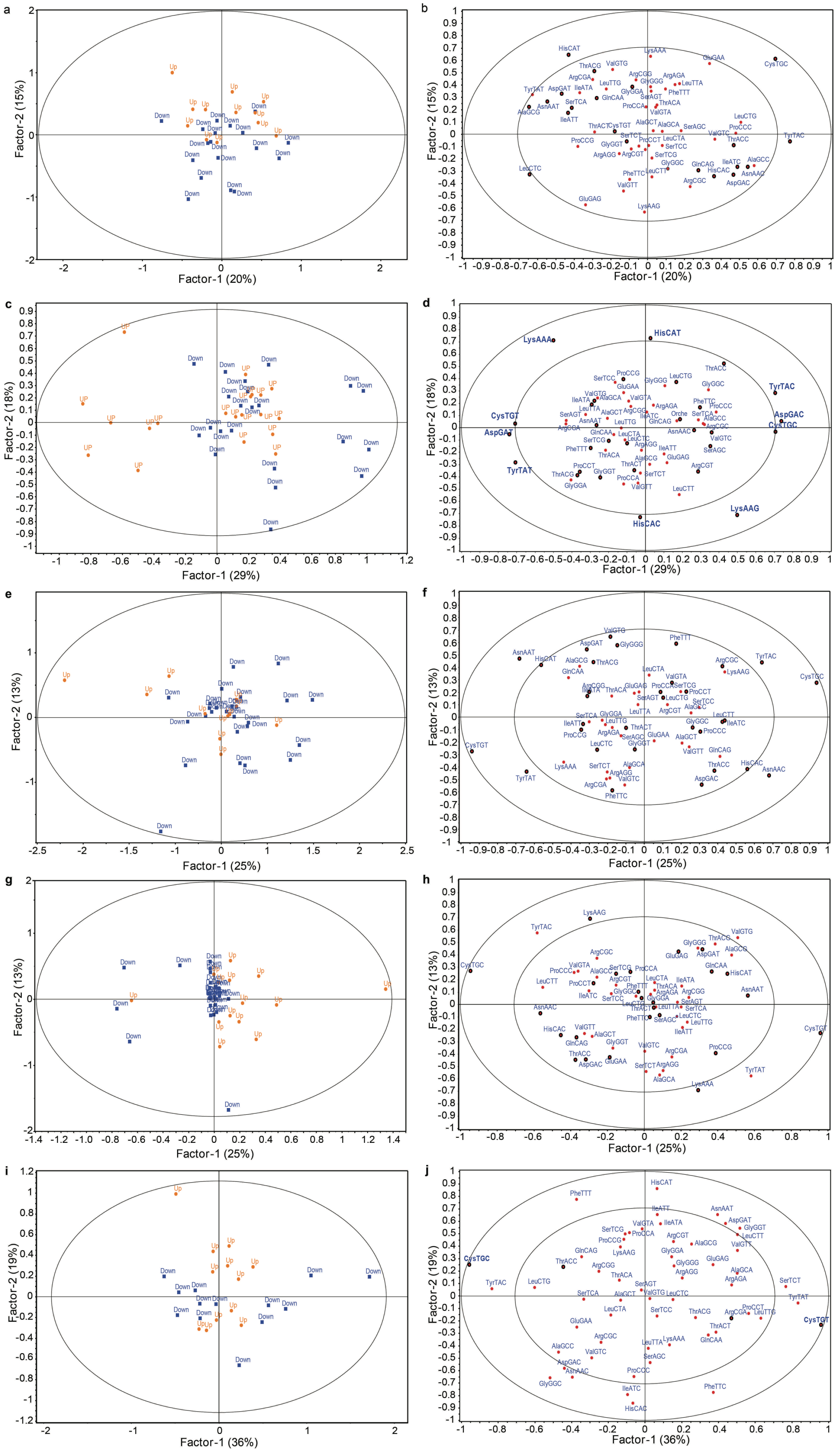

Supplementary Figure 7. Up- and down- regulated proteins during the entry and exit from hypoxia-induced bacteriostasis arise from genes with divergent codon usage patterns. PLS regression analysis of the most significantly up- (●) or down- (■) regulated proteins and their codon usages (●) visualized by scores: (**a**) H6; (**c**) H9; (**e**) H14; (**g**) H18; (**i**) R3); and corresponding loadings plots: (**b**) H6; (**d**) H9; (**f**) H14; (**h**) H18; (**j**) R3. Eclipse in scores plots represents Hoteling T^2 limit at p-value of 0.05 (F-test) while those in loadings plots indicate the explained variance. Outer and inner ellipses indicate 100% and 50% explained variance, respectively. Codons contributing significantly to the regression (cross validation, by applying Marten's Uncertainty Test) are circled in black. Percentages of observed variances explained by the Factor 1 and 2 are indicated in parentheses along their respective axes. In R6 vs. Log (**Supplementary Fig. 6g**), no proteins made the cut-off, so PLS regression analysis was not performed.

Supplementary Figure 8

a

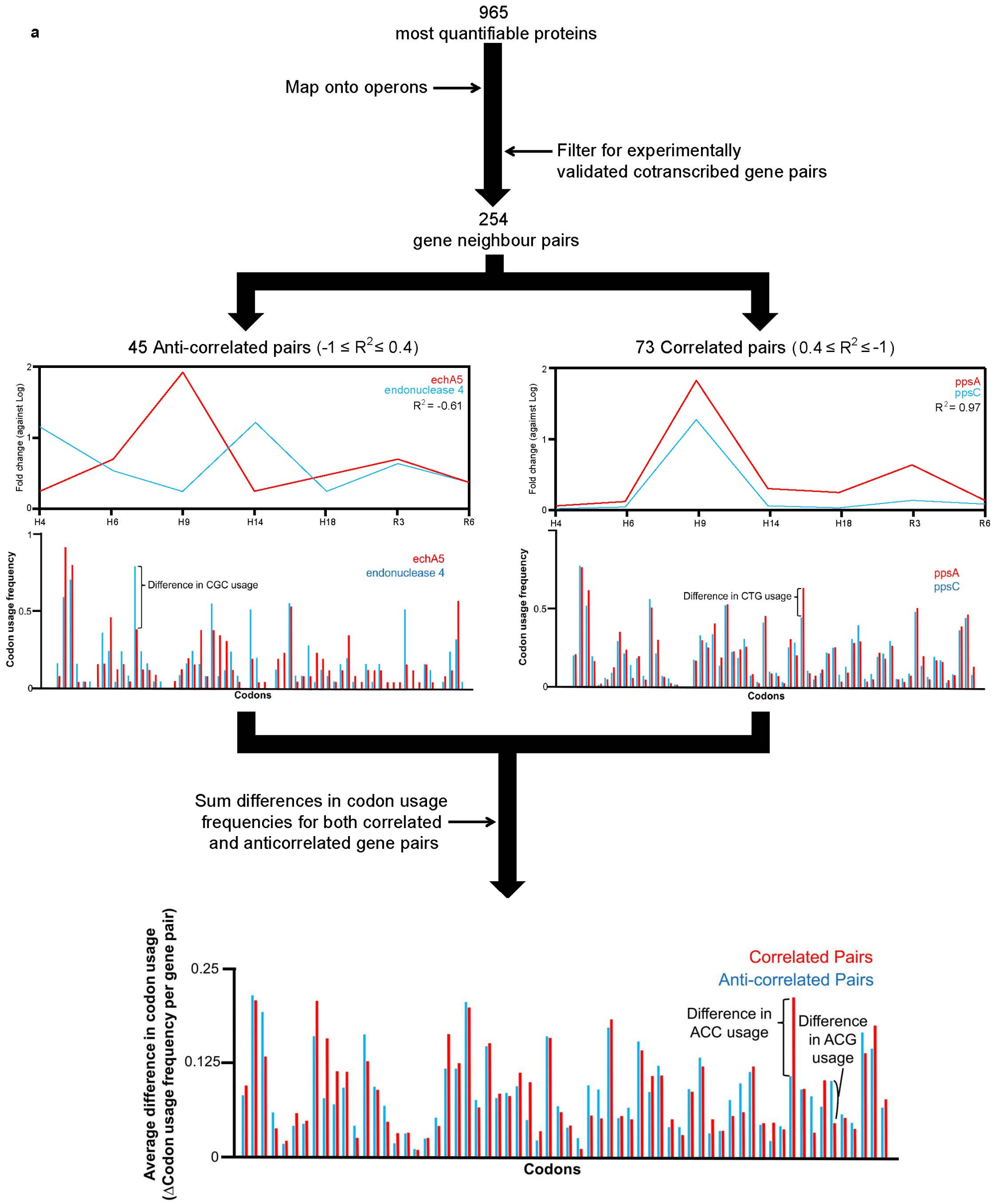

b

2 x 2 Contingency Table

	Anti-correlated pairs (-1 ≤ R^2 ≤ 0.4)	Non-anti-correlated pairs (0.4< R^2 ≤ 1)	Total
ACG preference in only 1 member of gene pair	29	42	71
No ACG preference in either gene <u>OR</u> ACG preference in both genes	16	167	183
**Total**	45	209	254

$$\text{Relative risk} = \frac{(29) / (29 + 42)}{(16) / (16 + 167)} = 4.7$$

Supplementary Figure 8. Codon usage influences on the differential expression of operon-encoded proteins. (**a**) Expression of 965 quantified proteins at Log, H4, H6, H9, H14, H18, R3 and R6 were analyzed based on operons. 229 genes can be mapped onto 86 operons which were experimentally validated to be co-transcribed with 2 or more genes in polycistronic mRNAs. 256 pair-wise comparisons of protein expression between co-transcribed genes were made. 73 pairs showed correlated protein expression ($R^2 \geq 0.4$) while 45 showed anti-correlated expression ($R^2 \leq -0.4$). Representative plots of changes in protein expression between operon-encoded pairs *echA5* (red) against *endonuclease 4* (blue) and *ppsA* (red) against *ppsC* (blue) are shown. Differences in codon usage frequencies between operon-encoded gene pairs are summed. Visualization as a histogram independent identification of ACC/ACG bias as one difference between correlated and anti-correlated pairs. (**b**) Relationship between protein expression correlation and ACG usage preference in gene pairs within co-transcribed operonic mRNA displayed as a 2-way contingency table. Numbers in each cell represent observed counts in each category. As 17.7% of the gene-pairs show anti-correlated protein expression ($R^2 \leq -0.4$), relative risk was calculated to determine the increased attributable likelihood of ACG preference in one member of an operonic gene-pair contributing to anti-correlated protein expression levels between gene-pair members. Relative risk = 4.6; 95% confidence interval 2.7 - 8.1; $z > 5.5$; $p < 0.0001$.

Supplementary Figure 9

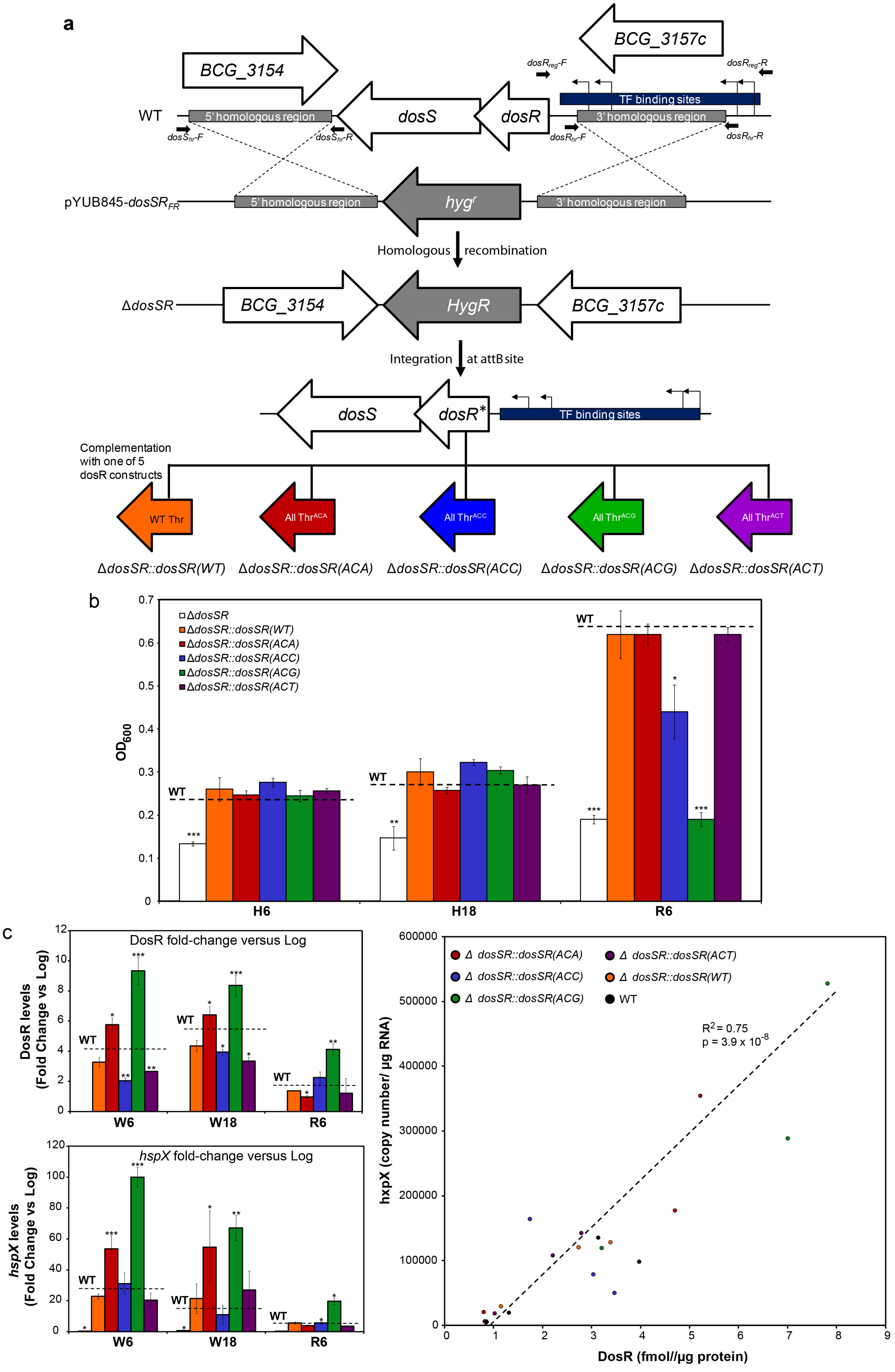

Supplementary Figure 9: *dosR* mutants, reengineered with altered threonine codon usage, possess varied fitness and mistimed DosR activity. (**a**) Construction of mutant BCG strains containing *dosR* with altered threonine codon usage. *dosSR* were first deleted. Subsequent complementation re-introduced reengineered *dosSR* constructs with its native promoter generating strains with genotypes described in **Supplementary Table 2**. See Methods for full description of the process. (**b**) Turbidity (OD_{600}) of cultures of recombinant BCG at H6, H18 and R6. Dashed line denotes the mean OD_{600} for WT cultures. (**c**) Fold-change in DosR (*top left*) and *hspX* (*bottom left*) expression of mutant strains relative to WT at H6, H18 and R6. Strength of the association between DosR protein levels and *hspX* mRNA induction estimated by linear regression (*right*). Regression statistics: Multiple R = 0.87, $R^2 = 0.75$, n = 24, F = 67.3, p = 3.89 x 10^{-8}. For (**b**) and (**c**), n ≥ 5; mean ± SEM; $p < 0.05$, $p < 0.01$ and $p < 0.001$ are denoted as *, ** and ***, respectively, determined by two-way ANOVA with Bonferroni post-tests (vs. Log) considering interactions between mutations and hypoxia. Together with changes in CFU (**Fig. 4a**) and DosR expression (**Supplementary Table 3**), choice of threonine codon contributes 29.9-42.7% to the total variance ($p < 0.0001$); hypoxic exposures 20.3-38.4% ($p < 0.0001$) and interactions between the two 21.6-22.7% ($p < 0.0001$).

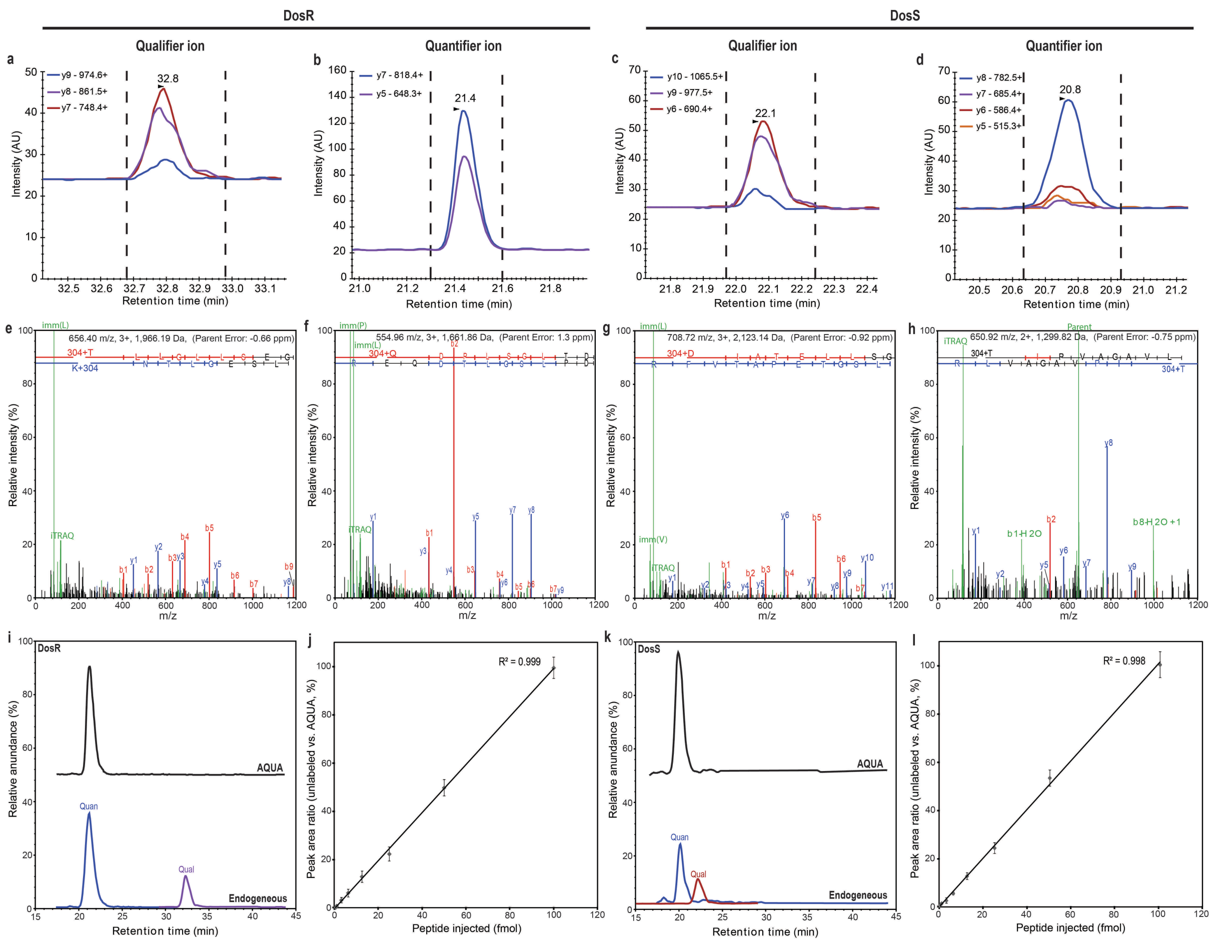

Supplementary Figure 10: Targeted LC-MS quantification of DosR and DosS protein. A two stage approach was used for SRM based targeted protein quantification: first method development, then, application to wild type and *dosR* mutants across the hypoxia-aerated recovery time course (**Supplementary Fig. 1a**). For method development, Skyline software was used to pick quantifier and qualifier ions against the BCG proteome background and the two best peptides, their fragments and SRM transitions selected (**a-c**). Each peptide and fragment was validated against the iTRAQ proteome MS/MS library (**e-h**). imm(L/P/V) are immonium ions of Leu, Pro and Val respectively. This method was used to determine DosR and DosS levels in wild-type and *dosR* mutants. Representative LC-SRM traces for 50 fmol of AQUA peptide and their corresponding endogenous counterparts for DosR (**i**) and DosS (**k**) respectively. Quan: quantifier ion, Qual: qualifier ion. Quantification was derived from the extracted ion chromatograms of unlabeled/labeled peptide pairs (QDPLSGLTDQER for DosR (**j**) and TIPVAGAVLR for DosS (**l**)). For both proteins, the estimated AQUA quantities in Δ*dosSR* BCG protein digests were plotted against the known spiked quantities of their unlabeled *in vitro* synthesized counterparts. DosR and DosS levels presented in **Supplementary Table 3**.

Supplementary Table 1. Quantification of DosR and DosS in *dosR* codon-engineered mutants

Strain		Concentration at Log (fmol/µg protein)	Fold-change at time point against Log				
			H6	H9	H14	H18	R6
**WT**	DosR	0.9 ± 0.2	3.7 ± 0.3	4.0 ± 0.4	5.4 ± 0.5	5.0 ± 0.4	1.5 ± 0.1
	DosS	1.1 ± 0.3	3.0 ± 0.2	3.0 ± 0.2	3.9 ± 0.3	5.3 ± 0.0	1.7 ± 0.1
Fold-change ratio (DosR/DosS)			*1.2*	*1.4*	*1.4*	*1.0*	*0.9*
ΔdosSR::dosSR(WT)	DosR	0.8 ± 0.2	3.3 ± 0.3	3.7 ± 0.3	4.8 ± 0.4	4.4 ± 0.4	1.4 ± 0.0
	DosS	1.2 ± 0.2	2.6 ± 0.2	2.9 ± 0.3	3.7 ± 0.3	3.7 ± 0.3	1.5 ± 0.2
Fold-change ratio (DosR/DosS)			*1.2*	*1.3*	*1.3*	*1.2*	*0.9*
ΔdosSR::dosSR(ACA)	DosR	0.8 ± 0.2	5.8 ± 0.4	8.6 ± 0.9	7.6 ± 0.6	6.4 ± 0.6	1.0 ± 0.1
	DosS	1.3 ± 0.2	5.2 ± 0.5	6.8 ± 0.6	6.1 ± 0.5	5.4 ± 0.4	1.1 ± 0.9
Fold-change ratio (DosR/DosS)			*1.1*	*1.3*	*1.2*	*1.2*	*0.9*
ΔdosSR::dosSR(ACC)	DosR	0.8 ± 0.2	2.0 ± 0.2	3.3 ± 0.3	4.8 ± 0.4	3.9 ± 0.2	2.3 ± 0.4
	DosS	1.3 ± 0.2	2.8 ± 0.3	4.4 ± 0.3	6.5 ± 0.6	5.2 ± 0.4	2.4 ± 0.4
Fold-change ratio (DosR/DosS)			*0.7*	*0.8*	*0.7*	*0.8*	*0.9*
ΔdosSR::dosSR(ACG)	DosR	0.8 ± 0.1	9.3 ± 0.9	12.1 ± 1.0	11.3 ± 1.0	8.4 ± 0.7	4.1 ± 0.4
	DosS	1.2 ± 0.2	5.8 ± 0.4	6.9 ± 0.6	8.0 ± 0.7	6.1 ± 0.5	3.2 ± 0.3
Fold-change ratio (DosR/DosS)			*1.6*	*1.8*	*1.4*	*1.4*	*1.3*
ΔdosSR::dosSR(ACT)	DosR	0.8 ± 0.1	2.7 ± 0.0	3.3 ± 0.3	3.7 ± 0.3	3.4 ± 0.3	1.2 ± 0.7
	DosS	1.3 ± 0.2	2.7 ± 0.2	2.7 ± 0.2	3.1 ± 0.2	3.1 ± 0.0	1.3 ± 0.1
Fold-change ratio (DosR/DosS)			*1.0*	*1.2*	*1.2*	*1.1*	*0.9*

Values presented with ± SEM. For WT, n = 6. For *dosR* mutants, n = 5.

Supplementary Table 2. Distribution of proteins from ACG-enriched among those that are up-regulated, down-regulated and unchanged at each point in the hypoxia time course.

Time		***Up-regulated***	***Down-regulated***	***No change***	***Total***
H4	ACG-biased	15	2	16	33
	All proteins	41	112	812	965
H6	ACG-biased	14	2	17	33
	All proteins	37	111	817	965
H9	ACG-biased	16	3	14	33
	All proteins	93	132	740	965
H14	ACG-biased	15	3	15	33
	All proteins	42	182	741	965
H18	ACG-biased	16	2	15	33
	All proteins	62	116	787	965
R3	ACG-biased	15	1	17	33
	All proteins	68	48	849	965
R6	ACG-biased	4	0	29	33
	All proteins	116	33	816	965

Up-reg'd: Log2-fold change >0 AND p <0.05; ***Down-reg'd***: Log2-fold change <0 AND p <0.05; ***Unchanged***: Log2-fold change=0 OR p>0.05; all changes vs. Log growth

Supplementary Table 3. Primer, oligonucleotide, peptide sequences, and strains.

Primers for cloning of 5’ and 3’ homologous regions

Name	Direction	Sequence (5’-3’)#	Tm (^{o}C)
$dosR_{hr}$	Forward	TTTCTAGAGCTGGTATGCACCGCACAAT	66.1
	Reverse	TTCTTAAGCCAGTAACGTACCGCTGAA	
$dosS_{hr}$	Forward	TTACTAGTCTCAGCTGTGGTGTGGCATT	65.4
	Reverse	TTCTCGAGCCGTTGTCGCAGTAGCTCTT	

Primers for cloning *dosSR* promoter and TF binding region

Name	Direction	Sequence (5’-3’)#	Tm (^{o}C)
$dosR_{reg}$	Forward	ttGTCGACcggcggatcggagattgat	65.1
	Reverse	ttAAGCTTcggcggatcggagattgatc	

Primers for screening of mutants

Gene	Direction	Sequence (5’-3’)	Tm (^{o}C)
dosR	Forward	GTGCCCTGGTGGTAAAGGTCT	61.0
	Reverse	GAGGCCCCCTGTTGTCATGG	
dosS	Forward	CGGTTCGGGTGCGCGATGAA	61.0
	Reverse	GGTGAAAGCCCCTGGACCGC	
*ureC**	Forward	TCGACCCCGGCGGCCATC	66.0
	Reverse	GGTGGACGGTCGGATCCG	
*hyg^{r}**	Forward	ACTGCTTGTCCGATATCTGAT	61.0
	Reverse	GAACTGGCGCAGTTCCTCT	

Primers for qPCR

Gene	Direction	Sequence (5’-3’)	Denaturing temperature (^{o}C)
dosR	Forward	AGGCGATGGCCAGGGTTCCT	90.0
	Reverse	GCGCGCGCCAACTCCATTCCCT	
dosS	Forward	CGGTTCGGGTGCGCGATGAA	92.0
	Reverse	ACAAGCCGGAACACCGTCGC	
hspX	Forward	GACGAGATGAAAGAGGGGCG	90.0
	Reverse	GTCGTCCTCGTCAGCACCTA	
sigA^	Forward	CGATGAGCCGGTAAAACGC	91.0
	Reverse	GAGCCACTAGCGGACTTCGC	

Oligonucleotides for the quantitation of tRNA species

tRNA	Role`	Sequence (5’-3’)	MRM Transition#
$tRNA_{CGU}$	Cal	rCrUrCrGrUrA	Quan: 916.1 → 346.1 (w1)<br>Qual: 916.1 → 572.1 (y2)
	IStd	CTCGUA	Quan: 882.2 → 330.1 (w1)<br>Qual: 882.2 → 634.1 (w2)
$tRNA_{GGU}$	Cal	rCrUrCrGrCrCrUrUrGrUrA	Quan: 929.1 → 346.1 (w1)<br>Qual: 929.1 → 344.0 (c1)
	IStd	CTCGCCTTGTA	Quan: 895.2 → 330.1 (w1)<br>Qual: 895.2 → 619.1 (c2)
$tRNA_{UGU}$	Cal	rGrCrGrUrUrUrCrCrA	Quan: 849.4 → 304.0 (c1)<br>Qual: 849.4 → 917.2 (y3)
	IStd	GCGTTTCCA	Quan: 819.4 → 303.0 (c1)<br>Qual: 819.4 → 634.1 (w2)

Peptides for the targeted quantitation of proteins

Protein	Role[~]	Sequence (N- to C- terminus)	SRM Transition[#]
DosR	Quan	QDPLSGLTDQER	679.8 → 818.4 (y7)
	Qual	TLLGLLSEGLTNK	679.9 → 861.6 (y8)
DosS	Quan	TIPVAGAVLR	498.8 → 782.5 (y8)
	Qual	DIATELLSGTEPATVFR	607.3 →690.4 (y6)
SigA	Quan	SVKPASAPQDTTTSTIPK	686.15 → 548.4 (y2)
	Qual	KDAELTASADSVR	657.7 → 361.2 (y3)

Strains and characteristics

Strain	Genotype
BCG Pasteur 1173P2	Wild-type (WT)
Δ*dosSR*	As 1173P2 plus Δ(*dosS-dosR)*::*hyR*
Δ*dosSR*::*dosSR(WT)*	As Δ*dosSR* plus *attB*::[pMV306::*dosS-dosR*]::*kan*
Δ*dosSR*::*dosSR(ACA)*	As Δ*dosSR* plus *attB*::[pMV306::*dosS-dosR*(40C>T + 61C>T + 115C>T +157G>T + 187C>T + 202G>T + 400G>T + 409C>T]::*kan*
Δ*dosSR*::*dosSR(ACC)*	As Δ*dosSR* plus *attB*::[pMV306::*dosS-dosR*(40C>G + 61C>G + 115C>G + 187C>G + 409C>G]::*kan*
Δ*dosSR*::*dosSR(ACG)*	As Δ*dosSR* plus *attB*::[pMV306::*dosS-dosR*(157G>C + 202G>C+ 400G>C]::*kan*
Δ*dosSR*::*dosSR(ACT)*	As Δ*dosSR* plus *attB*::[pMV306::*dosS-dosR*(40C>A + 61C>A+ 115C>A +157G>A + 187C>A + 202G>A + 400G>A + 409C>A]::*kan*

[#] Restriction endonuclease sites underlined. * Positive controls. Sequences obtained from Ref. 20. [^]Sequences Obtained from Ref. 21. `Cal: External calibration standards. IStd: Spiked internal standards used for relative quantitation. # Quan: Quantifier ion. Qual: Qualifier ion. Fragment identity indicated in parentheses. ~ Quan: Quantifier peptide. Qual: Qualifier peptide. Unique peptide sequences from tryptic digests of target proteins.

Supplementary Method 1

Reagents. Unless otherwise stated, chemical reagents were purchased from Sigma-Aldrich, bacterial culture reagents from BD Biosciences, Purelink RNA extraction kits were purchased from Life Technologies, LC-MS grade solvents from Thermo-Fisher, RNA and DNA oligonucleotides from Integrated DNA Technologies, restriction enzymes from New England Biolabs, AQUA peptides from Sigma Aldrich and unlabeled peptide standards from Genescript.

Bacterial cultures. For mycobacteria pre-cultures, *Mycobacterium bovis* Bacille Calmette-Guérin (str. Pasteur 1173P2; BCG) was grown at 37 ^{o}C in a shaking incubator in Middlebrook 7H9 broth supplemented with 0.5% (w/v) albumin, 0.2% (w/v) glucose, 0.085% (w/v) NaCl, 0.2% (v/v) glycerol and 0.05% (v/v) Tween 80 to an OD_{600} of 0.6 – 0.8. For exponential growth, BCG pre-cultures were inoculated into Dubos broth (supplemented with 10% Dubos medium albumin, and 0.03% Tween 80) and passaged in roller bottles for balanced growth. A non-replicating state was induced in BCG by subjecting them to the slow withdrawal of oxygen as described by Wayne and Hayes [22] with modifications made by Ravindran *et al.*[1] for a ~40-fold expansion of batch cultures. Briefly, a culture of exponentially growing BCG in Dubos broth was diluted to OD_{600} 0.005 and placed in a tightly-sealed (latex-lined cap) 1 L glass flask (Duran) with stirring at 80 rpm and an air headspace ratio of 0.5. For resuscitation of dormant cultures by re-aeration, hypoxic cultures were transferred to 1.5 L vent-capped Erlenmeyer flasks (Corning) and shaken at 180 rpm. Levels of oxygen were tracked by methylene blue decolorization, oxygen indicator strips (BD) and dissolved oxygen probe sensor (Vernier). Cultures with added methylene blue were not used for any analysis other than oxygen level. At indicated time points (**Extended Data Fig. 1**), flasks were sacrificially opened and the bacteria were pelleted, washed, and flash-frozen in liquid nitrogen. For CFU determinations, serial dilutions were plated on 7H11 agar supplemented with 10% (v/v) oleic acid-albumin-dextrose-catalase (OADC). Cultures were routinely assessed for contamination by microscopy (Gram, Ziehl-Neelsen, and auramine-rhodamine staining) and streaking on blood agar. When appropriate, hygromycin and kanamycin were added at 80 and 30 µg/mL, respectively, to broth and agar. *Escherichia coli* (DH10β/TOP10) were grown in Luria-Bertani (LB) broth and agar. Where appropriate, hygromycin and kanamycin were added to 150 and 50 µg/mL, respectively.

Supplementary Method 2

RNA extraction and purification. Total RNA was extracted from BCG pellets as described previously[23]. To prevent the formation of Tris-RNA adducts[24], TRIzol (Life Technologies) instead of Tris-EDTA buffer saturated phenol/chloroform/isoamyl alcohol was used with bead beating for cell lysis. Additionally, on-column DNase I (Qiagen) digestion was performed in the presence of 10 mM ammonium acetate (pH 7.0) instead of Tris-buffer. Multi-dimensional HPLC for isolation and quantification of RNA species was performed as previously described[25]. RNA composition and integrity was determined using the appropriate Agilent Bioanalyzer RNA chips. Total RNA samples with a RNA Integrity Number (RIN) of 8.0 or greater were used for qPCR experiments. tRNA samples that were >99.9% pure by size-exclusion chromatography were used for LC-MS/MS analysis.

Data reduction and signal processing for oligonucleotide mapping. Agilent MassHunter software BioConfirm Suite was used to perform molecular feature extraction (MFE), spectra deconvolution, de-isotoping[26], and background subtraction for both MS (maximum entropy) and

MSMS (resolved isotope) spectra deconvolution. A conservative background threshold of 200 spectra counts and MFE score of greater than 80 as our cutoff values for inclusion. Spectra intensities were sum across the entire LC elution peak. As the software currently do not support direct analysis of RNA modifications, we exported the deconvoluted molecular ion spectra (z= -1) of each singular molecular ion species separately for further analysis.

De novo sequencing and soring of oligonucleotide mass spectra. We used RoboOligo (https://u.osu.edu/paulsample/robooligo) automated local search approach algorithm to build sequences from deconvoluted spectra one nucleotide at a time after "oligonucleotide fitness" was evaluated by the abundance of c- and y- ions. Potential sequences were scored based on summing the abundances at each set of observed product ions to produce a cumulative score. In all cases, possible sequences were ranked and the hits matched to sequences derived from theoretical digests of tRNA-coding genomic DNA.

Validation of oligonucleotide sequences and modification assignments. Unmodified RNA oligonucleotides from the predicted RNAse U2 digest sequences were used as standards for comparison of fragmentation products on the LC-QToF. Unmodified synthetic oligonucleotides that eluted more than 1 min away from the targeted modified oligonucleotide of interest were eliminated as the possible unmodified precursor of the modified oligonucleotide. MSMS spectra for both modified oligonucleotide and unmodified standards were then analyzed by SOS (http://mods.rna.albany.edu/Masspec-Toolbox/SOS). MSMS spectra of the modified ribonucleotide residue were considered to have confirmed identities when there are mass shifts in two or more assigned c, y, w and a-B ions that matches are attributed to the same modification.

Supplementary Method 3

Protein extraction and processing. Hexaplicate samples of BCG pellets at 8 experimental time points (Log, H4, H6, H9, H14, H18, R3 and R6) were extracted with 8 M urea, 1 M thiourea with 2% SDS supplemented with protease inhibitor complete cocktail (Roche) by bead beating with 0.2 mm silica beads in a TissueLyzer II (Qiagen) operated at 50 Hz for three 5-min cycles. Bead beating chambers were chilled at -20 °C before and between cycles. Extracts were centrifuged at 14,000 g for 15 min at 4 °C and the supernatant collected. Supernatants were diluted with equal volumes of ultra-pure water and supplemented with HALT protease inhibitor cocktail (Pierce BioSciences), $MgCl_2$ (2 mM final) and Tris-HCl (pH 8.0, 10 mM final) for Benzonase digestion (25 Units per mL lysate) at 20 °C for 3 h. Samples were chilled on ice and sonicated (20% maximum amplitude, five 10-s cycles, Fisher-Scientific FB15061) to break up any aggregates that might had formed. Overnight trichloroacetic acid/acetone precipitation was performed with 2D Clean-Up kits (GE Healthcare) as instructed by the manufacturer. Air-dried protein pellets were resuspended in 10 mM triethylammonium bicarbonate (TEAB) buffer (pH 8.5) and protein concentrations determined by BCA assay (Pierce BioSciences). Protein quality and quantities was checked by SDS-PAGE electrophoresis (12% polyacrylamide gels) and purity by UV spectrometry (Nanodrop, Thermo Scientific). Protein aliquots (100 µg) were collected, lyophilized, and stored at –80 °C prior to further processing.

Lyophilized proteins were reconstituted in 100 mM TEAB and 10% acetonitrile (v/v) by bath sonication. Samples were reduced and alkylated using the iTRAQ kit (Applied Biosystems) according to the manufacturer's instructions. Protein concentrations were estimated using Bradford assays (Bio-Rad). 50 µg of protein was precipitated by cold acetone, redissolved in 6 M

urea, diluted with five volumes of 100 mM TEAB with 10% (v/v) acetonitrile and digested with trypsin in a 1:30 (w/w) ratio overnight at 37 °C. Samples were dried by vacuum centrifugation and stored at –20 °C prior to analysis.

Proteomics data processing and database searching. Tandem mass spectra were extracted, charge state deconvoluted and deisotoped by Spectra Mill (Agilent; v B.04.00.127). All MS/MS samples were analyzed using Spectrum Mill and X!Tandem (The GPM, thegpm.org; version CYCLONE (2010.12.01.1)). Spectrum Mill and X!Tandem were set up to search the SwissProt.BCG.Pasteur.1173P2 database (selected for all curated and non-curated proteins. Retrieved Apr 27 2014, 3891 entries) with tryptic digest fragments with an ion mass tolerance of 50 PPM and a parent ion tolerance of 20 PPM. Carbamidomethyl of cysteine and iTRAQ 8plex of lysine and the n-terminus were specified in Spectrum Mill and X!Tandem as fixed modifications. Ammonia-loss of the N-terminus, deamidated of asparagine and oxidation of methionine were specified in Spectrum Mill as variable modifications. Glu->pyro-Glu of the n-terminus, ammonia-loss of the N-terminus, gln->pyro-Glu of the N-terminus, deamidated of asparagine, oxidation of methionine, acetyl of lysine, carbamidomethyl of cysteine, phosphorylation of serine, threonine and tyrosine and iTRAQ8plex of lysine and the N-terminus were specified in X!Tandem as variable modifications.

Criteria for protein identification. Scaffold (version Scaffold_4.3.0, Proteome Software Inc.) was used to validate MS/MS based peptide and protein identifications. Peptide identifications were accepted if they could be established at greater than 95% probability by the Scaffold Local FDR algorithm. Protein identifications were accepted if they could be established at greater than 95% probability and contained at least 2 identified peptides. Protein probabilities were assigned by the Protein Prophet algorithm[27]. Proteins that contained similar peptides and could not be differentiated based on MS/MS analysis alone were grouped to satisfy the principles of parsimony. Proteins sharing significant peptide evidence were grouped into clusters. Annotated with GO terms from 25994.M_bovis_Pasteur_1173P2.goa (downloaded from NCBI May 5, 2014) was performed[28].

Relative protein quantification by iTRAQ. Scaffold Q+ (version Scaffold_4.3.4, Proteome Software Inc.) was used to quantify iTRAQ Label Based Quantitation peptide and protein identifications. Peptide identifications were accepted if they could be established at greater than 90.0% probability by the Scaffold Local FDR algorithm. Protein identifications were accepted if they could be established at greater than 99% probability and contained at least 2 identified peptides. Protein probabilities were assigned by the Protein Prophet algorithm[27]. Proteins sharing significant peptide evidence were grouped into clusters. Channels were corrected by the matrix [0.000,0.000,0.929,0.0689,0.00220]; [0.000,0.00940,0.930,0.0590,0.00160]; [0.000,0.0188,0.931,0.0490,0.001000]; [0.000,0.0282,0.932,0.0390,0.000700]; [0.000600,0.0377,0.933,0.0288,0.000]; [0.000900,0.0471,0.933,0.0188,0.000]; [0.00140,0.0566,0.933,0.00870,0.000]; [0.000,0.000,0.000,0.000,0.000]; [0.00270,0.0744,0.921,0.00180,0.000] in all samples according to the i-Tracker algorithm[29]. Acquired intensities in the experiment were globally normalized across all acquisition runs. Individual quantitative samples were normalized within each acquisition run. Intensities for each peptide identified were normalized within the assigned protein. The reference channels were normalized to produce a 1:1 fold change. All quantitative calculations were performed using means of the multiplicatively normalized data. Differentially expressed proteins were determined using one-way ANOVA using Bonferroni multiple testing correction.

Supplementary Method 4

Software settings for multivariate statistical analysis. Differential abundance of tRNAs, tRNA modifications and proteins was analyzed by a random effects Bayes model using the BETR algorithm in MeV (http://www.tm4.org/mev.html). Percentages of modified tRNA species are determined by peak area normalization with response factors. Clustering analysis was performed using two-way hierarchical clustering with average distances and complete linkages and visualized using MeV. Shifts in the BCG proteome were analyzed by principle component analysis (PCA) using the NIPALS algorithm. Interpretations of the relationships between codon usage predictors (codon frequency) and protein up-or down- regulation ($\log_2$ median fold change) were analyzed by partial least squares regression (PLS). Alternate start and stop codons were treated as categorical variables. Outliers that could cause over-fitting were removed by inspection of variable residuals and leverages. Validation was performed using cross-validation and the significance of variables determined by Marten's uncertainty test. The Root Mean Square Error of Prediction (RMSEP), slope and correlation coefficient of predicted versus measured correlation line was used to evaluate the efficiency of applied regression model. Eigenvector based multivariate statistics performed using UnscramblerX® (v10.3, Camo).

Supplementary References

1. Ravindran, M. S. *et al.* Targeting lipid esterases in mycobacteria grown under different physiological conditions using activity-based profiling with tetrahydrolipstatin (THL). *Mol Cell Proteomics* **13**, 435-448, (2014).
2. Boon, C. & Dick, T. How Mycobacterium tuberculosis goes to sleep: the dormancy survival regulator DosR a decade later. *Future Microbiol* **7**, 513-518, (2012).
3. Kumar, A., Toledo, J. C., Patel, R. P., Lancaster, J. R., Jr. & Steyn, A. J. Mycobacterium tuberculosis DosS is a redox sensor and DosT is a hypoxia sensor. *Proc Natl Acad Sci U S A* **104**, 11568-11573, (2007).
4. Rustad, T. R., Harrell, M. I., Liao, R. & Sherman, D. R. The enduring hypoxic response of Mycobacterium tuberculosis. *PLoS One* **3**, e1502, (2008).
5. Galagan, J. E. *et al.* The Mycobacterium tuberculosis regulatory network and hypoxia. *Nature* **499**, 178-183, (2013).
6. Selvaraj, S., Sambandam, V., Sardar, D. & Anishetty, S. In silico analysis of DosR regulon proteins of Mycobacterium tuberculosis. *Gene* **506**, 233-241, (2012).
7. Tumu, S., Patil, A., Towns, W., Dyavaiah, M. & Begley, T. The gene-specific codon counting database: a genome-based catalog of one-, two-, three-, four- and five-codon combinations present in *Saccharomyces cerevisiae* genes. *Database* **2012**, bas002, (2012).
8. Bjork, G. R., Huang, B., Persson, O. P. & Bystrom, A. S. A conserved modified wobble nucleoside (mcm5s2U) in lysyl-tRNA is required for viability in yeast. *RNA* **13**, 1245-1255, (2007).
9. Nasvall, S. J., Chen, P. & Bjork, G. R. The wobble hypothesis revisited: uridine-5-oxyacetic acid is critical for reading of G-ending codons. *RNA* **13**, 2151-2164, (2007).
10. Sorensen, M. A. *et al.* Over expression of a tRNA(Leu) isoacceptor changes charging pattern of leucine tRNAs and reveals new codon reading. *J Mol Biol* **354**, 16-24, (2005).
11. Takai, K. *et al.* Recognition of UUN codons by two leucine tRNA species from Escherichia coli. *FEBS Lett* **344**, 31-34, doi:0014-5793(94)00354-8 [pii] (1994).
12. Murphy, F. V. t., Ramakrishnan, V., Malkiewicz, A. & Agris, P. F. The role of modifications in codon discrimination by tRNA(Lys)UUU. *Nat Struct Mol Biol* **11**, 1186-1191, (2004).
13. Rodriguez-Hernandez, A. *et al.* Structural and mechanistic basis for enhanced translational efficiency by 2-thiouridine at the tRNA anticodon wobble position. *J Mol Biol* **425**, 3888-3906, (2013).
14. Yarian, C. *et al.* Accurate translation of the genetic code depends on tRNA modified nucleosides. *J Biol Chem* **277**, 16391-16395, (2002).
15. Crick, F. H. Codon--anticodon pairing: the wobble hypothesis. *J Mol Biol* **19**, 548-555 (1966).
16. Yokoyama, S. & Nishimura, S. in *tRNA: Structure, Biosynthesis and Function* (eds D. Soll & U. L. Rajbhandary) 207-223 (American Society for Microbiology, 1995).
17. Agris, P. F., Vendeix, F. A. & Graham, W. D. tRNA's wobble decoding of the genome: 40 years of modification. *J Mol Biol* **366**, 1-13, (2007).
18. Grosjean, H., de Crecy-Lagard, V. & Marck, C. Deciphering synonymous codons in the three domains of life: co-evolution with specific tRNA modification enzymes. *FEBS Lett* **584**, 252-264, (2010).

19 Roth, A. C. Decoding properties of tRNA leave a detectable signal in codon usage bias. *Bioinformatics* **28**, i340-i348, (2012).

20 Lin, W. *et al.* Urease activity represents an alternative pathway for Mycobacterium tuberculosis nitrogen metabolism. *Infect Immun* **80**, 2771-2779, (2012).

21 Volpe, E. *et al.* Gene expression profiling of human macrophages at late time of infection with Mycobacterium tuberculosis. *Immunology* **118**, 449-460, (2006).

22 Wayne, L. G. & Hayes, L. G. An in vitro model for sequential study of shiftdown of Mycobacterium tuberculosis through two stages of nonreplicating persistence. *Infect Immun* **64**, 2062-2069 (1996).

23 Hia, F. *et al.* Mycobacterial RNA isolation optimized for non-coding RNA: high fidelity isolation of 5S rRNA from Mycobacterium bovis BCG reveals novel post-transcriptional processing and a complete spectrum of modified ribonucleosides. *Nucleic Acids Res* **43**, e32, (2014).

24 Miyauchi, K., Kimura, S. & Suzuki, T. A cyclic form of N6-threonylcarbamoyladenosine as a widely distributed tRNA hypermodification. *Nat Chem Biol* **9**, 105-111, (2013).

25 Chionh, Y. H. *et al.* A multidimensional platform for the purification of non-coding RNA species. *Nucleic Acids Res* **41**, e168, (2013).

26 Wetzel, C., Li, S. & Limbach, P. A. Metabolic de-isotoping for improved LC-MS characterization of modified RNAs. *J Am Soc Mass Spectrom* **25**, 1114-1123, (2014).

27 Nesvizhskii, A. I., Keller, A., Kolker, E. & Aebersold, R. A statistical model for identifying proteins by tandem mass spectrometry. *Anal Chem* **75**, 4646-4658 (2003).

28 Ashburner, M. *et al.* Gene ontology: tool for the unification of biology. The Gene Ontology Consortium. *Nat Genet* **25**, 25-29, (2000).

29 Shadforth, I. P., Dunkley, T. P., Lilley, K. S. & Bessant, C. i-Tracker: for quantitative proteomics using iTRAQ. *BMC Genomics* **6**, 145, (2005).
